# Center of mass kinematic reconstruction during steady-state walking using optimized template models

**DOI:** 10.1371/journal.pone.0313156

**Published:** 2024-11-05

**Authors:** David J. Kelly, Patrick M. Wensing

**Affiliations:** Aerospace & Mechanical Engineering Department, University of Notre Dame, Notre Dame, Indiana, United States of America; University of Tehran, IRAN, ISLAMIC REPUBLIC OF

## Abstract

Template models, such as the Bipedal Spring-Loaded Inverted Pendulum and the Virtual Pivot Point, have been widely used as low-dimensional representations of the complex dynamics in legged locomotion. Despite their ability to qualitatively match human walking characteristics like M-shaped ground reaction force (GRF) profiles, they often exhibit discrepancies when compared to experimental data, notably in overestimating vertical center of mass (CoM) displacement and underestimating gait event timings (touchdown/ liftoff). This paper hypothesizes that the constant leg stiffness of these models explains the majority of these discrepancies. The study systematically investigates the impact of stiffness variations on the fidelity of model fittings to human data, where an optimization framework is employed to identify optimal leg stiffness trajectories. The study also quantifies the effects of stiffness variations on salient characteristics of human walking (GRF profiles and gait event timing). The optimization framework was applied to 24 subjects walking at 40% to 145% preferred walking speed (PWS). The findings reveal that despite only modifying ground forces in one direction, variable leg stiffness models exhibited a >80% reduction in CoM error across both the B-SLIP and VPP models, while also improving prediction of human GRF profiles. However, the accuracy of gait event timing did not consistently show improvement across all conditions. The resulting stiffness profiles mimic walking characteristics of ankle push-off during double support and reduced CoM vaulting during single support.

## Introduction

Walking is a basic function of legged locomotion that, at a fundamental level, can be viewed as the transportation of the center of mass (CoM) via the coordinated transfer of weight between the legs. Research has extensively focused on understanding the role of the CoM in locomotion [1], exploring its interaction with the base of support [2], its relationship with stability in challenging environments [3], and its role in motor feedback for both balance recovery [4] and maneuverability [5]. However, the complexity of the human body, with its numerous degrees of freedom and redundancy, complicates efforts to model the CoM’s evolution. To address this issue, a wide body of research has explored describing walking dynamics through the lens of template models [6].

Template models are low-dimensional dynamical systems for describing the evolution of system-level goals. In the case of legged locomotion, pendular systems have been a prime candidate for investigating characteristics such as CoM trajectories [7–10], ground reaction force (GRF) profiles [8, 9], and system energetics [7, 8, 11] throughout a gait cycle. By modeling legged locomotion via a mass supported on inverted pendulum(s), much of the high-dimensional complexity from the biomechanical interplay of muscles, tendons, bones, and joints can be stripped away without losing the salient system-level characteristics. Early examples demonstrated that modeling a mass on top of a massless spring leg, as in spring-loaded inverted pendulum (SLIP) models, could describe GRF and CoM energy profiles [7] for general human hopping and running. By incorporating a second spring leg, the descriptive capabilites of the model were extended to bipedal walking [8].

Beyond humans, SLIP-based models have been used to describe other legged animals. Spring-mass passive dynamics have been mapped across speed and size, predicting the mechanics of locomotion by scaling the leg stiffness with size and weight from birds like the guinea fowl [12], up to larger animals like kangaroos [13]. Even for leg contact sequences that differ from bipedal locomotion, like the quadrupedal gait of dogs or the three-leg contact of arthropods, energy management can be modeled by a single spring leg while retaining some observable qualities such as force oscillations [6, 13–16]. The significantly different musculoskeletal structures across these species [12, 17] highlights the ability of SLIP-based models to transcend across the biomechanical complexities of legged locomotion.

The low-dimensionality of these SLIP-based models also makes them tractable to include other desired characteristics without overcomplicating the underlying dynamics. Attempts to incorporate non-conservative energetics into SLIP-based models have been sought to better align the models with the non-conservative nature of legged locomotion [18]. These extensions have been accomplished via the inclusion of dampers [19–21] or by changing the rest leg length [22]. SLIP-based models have also been studied in 3-D to capture information like step width, CoM sway, and lateral GRF information [22–24]. Moving beyond point-mass assumptions, the incorporation of trunk dynamics has helped define upper-body stability paradigms through the use of hip torques [25] and redirection of GRFs to a point above the CoM [26–28]. Note that these extensions of SLIP-based models have also been experimentally observed across legged species [27, 29, 30]. Extending beyond the model itself, the robustness and disturbance rejection of SLIP-based models has been studied for uneven terrain [31], as well as different compliant surfaces [13, 32–34]. The latter have demonstrated that leg stiffness adjustments based on energy maintenance and injury prevention as the surface compliance changes from step-to-step can be captured via SLIP-based models.

Despite the body of work on understanding the dynamical properties of many common templates, comparative analysis with experimental CoM kinematics has been largely qualitative. Quantitative comparisons to experimental data has primarily focused on prediction of GRF profiles [19, 21, 35–37], but that has not necessarily translated to good quantitative matching of CoM trajectories. SLIP-based models often overestimate the vertical displacement of the CoM and underestimate gait event timings observed in human data [9]. Methods for selecting the parameters of these models also often focus on achieving stable gaits that are periodic and symmetric [8, 27], which may not necessarily represent legged locomotion [1, 11].

Extending these SLIP-based models to achieve quantifiably close fits to experimental CoM trajectories opens the door to several applications, particularly in physical human-robot interaction for lower-extremity assistive devices. Pendular models have been used extensively in the control of and design of legged robotics, from single [38, 39], to bipedal [22, 24, 40–43] and multi-legged [44–46]. However, legged robotics can be designed around desired outcomes and simulations, whereas the inverse should be considered in the context of human locomotion. In a human-robot locomotion interface, such as prostheses [47–50] and exoskeletons [51–53], the robotic device should work in conjunction with the human, as opposed to forcing the human to adapt, further motivating the improvement of quantifiably matching experimental CoM kinematics.

It is hypothesized that relaxing the constant spring stiffness used in SLIP-based models, and allowing the leg stiffness to vary throughout the stance phase of walking will account for the discrepancies between SLIP-based models and experimental walking data previously noted. More specifically, this work looks to *quantify* the impact of imposing a constant spring stiffness on the ability of these models to match a wide range of human data.

This methodology is inspired by how the activity level of a muscle changes its mechanical stiffness [54]. It has been experimentally observed that different lower-limb muscles are active at different phases of the gait cycle [55], and muscle activity increases at varying rates across lower-limb muscles as gait speed increases for humans [56]. The interplay between the timing of muscle activation and joint configuration of the lower-limb throughout the gait cycle complicates the relationship between joint quasi-stiffness (torque-angle relationship of a joint) and effective leg stiffness (force over the effective distance between the center of pressure and CoM). While ankle and hip joint quasi-stiffness experimentally increase with gait speed, effective leg stiffness tends to decrease for walking [9, 57–59] and remain constant for running [9, 60]. One potential source for this observation could arise from the use of one single value to define a joint’s stiffness, even though joints exhibit multiple quasi-stiffness regions based on angle displacement and phase of the gait cycle [58, 59, 61–63]. This relationship also arises because effective leg stiffness is typically only reported as the value of maximum force over maximum displacement for an entire stance phase [9, 57]. However, like joint quasi-stiffness, the force-displacement relationship during walking is not constant, varying by as much as three-fold throughout the gait cycle [64]. From this perspective, varying leg stiffness *within a stride* may better align with dynamic interplay created by the musculoskeletal coordination of muscle activation and joint displacement, and more accurately capture within-stride dynamics.

Allowing the leg spring stiffness to vary throughout the stance phase effectively provides an extra parameter to tune from a modeling perspective, which naturally should improve the ability of these models to match experimental data. Although the ground forces in our model are 2-D, we are interested in investigating how much of the CoM error can be accounted for by changing this one-dimensional stiffness parameter alone. While varying stiffness has been studied previously [65, 66], the focus of that work was towards the control of bipedal robots, as opposed to characterizing human locomotion herein.

**Fig 1:**
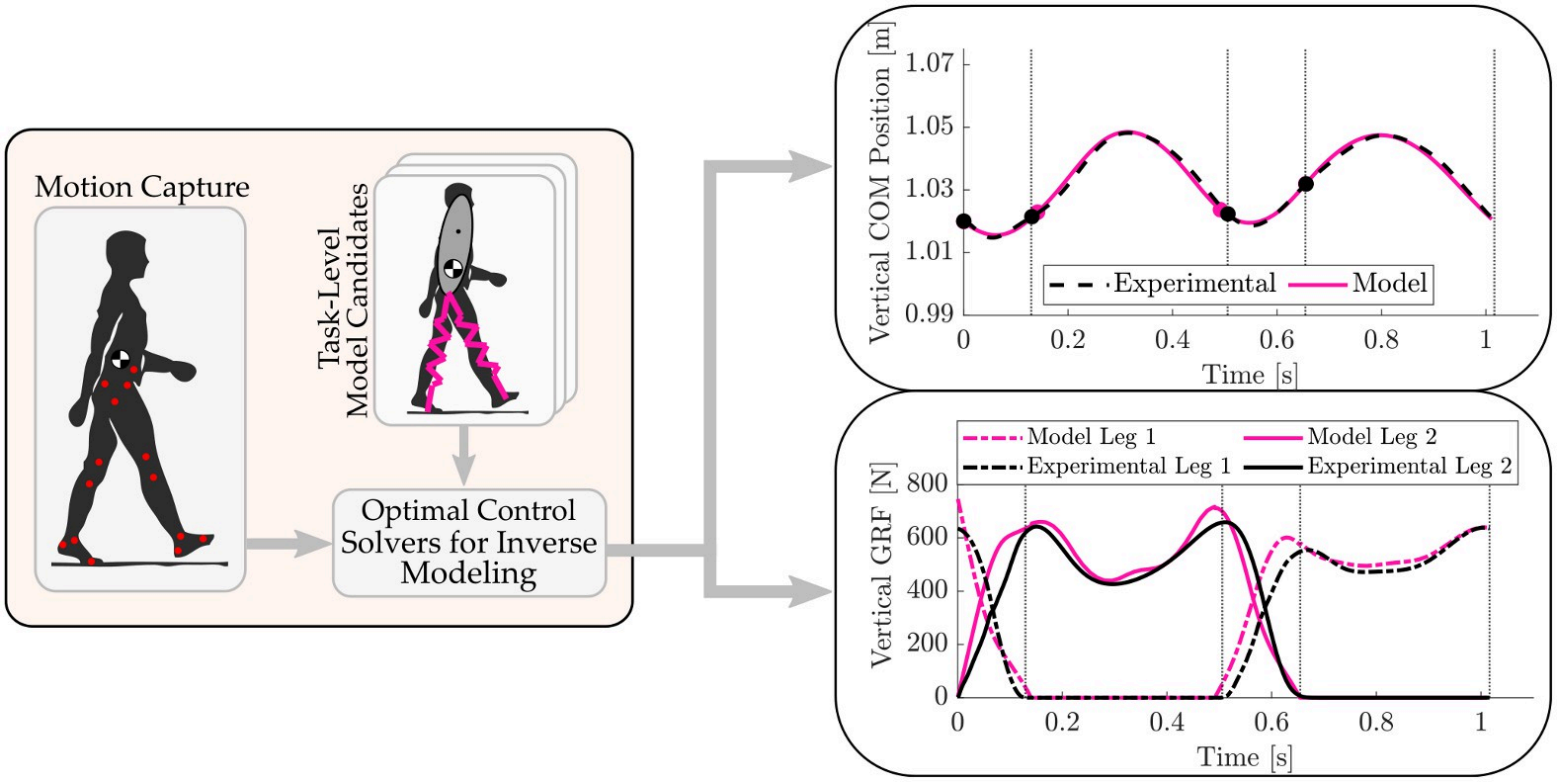
Workflow schematic. Trajectory optimization is used to determine the parameters that achieve accurate tracking of human walking data with template models.

To evaluate the limits of the constant stiffness assumption in predicting CoM kinematics, we hypothesize that SLIP-based models will show statistically significant improvement in matching CoM trajectories when leg stiffness is allowed to vary (H1). We further hypothesize that these improvements will extend to better matching GRF profiles (H2) and gait event timings (H3). Using an optimization framework with SLIP-based models, we determine the model parameters that best fit human walking CoM data (Fig 1). For each trial, both constant and varying stiffness versions of each model are optimized to assess the impact of the constant stiffness assumption. Quantitative metrics are designed to evaluate how well each model variation matches CoM position, GRF profiles, and gait event timings, and to analyze where in the gait cycle the constant stiffness assumption fails.

The rest of this paper is organized as follows. The data processing of the experimental data used in this work, as well as the formulation for the model dynamics and optimization framework are outlined next in the Methods section. The outcomes of applying the optimization framework to different variations of the template models are presented in the subsequent Results section. Examination of how the outcomes from this work fit into existing literature are explored in the Discussion. This section also includes a brief commentary on applying this work for control of a lower-limb powered prosthesis, before then providing concluding remarks.

## Methods

### Human data

The optimization framework was tested using a public data set of subjects walking on a treadmill [67]. The data included 24 young healthy individuals walking at 8 different treadmill speeds based on percentages of their preferred walking speeds (PWS) (40%, 55%, 70%, 85%, 100%, 115%, 130%, 145%). The lower extremity kinematic data for the subjects was obtained with a motion capture system at a rate of 150 Hz (Raptor-4 cameras). GRF information was obtained from Optima force plates integrated into the treadmill at a rate of 300 Hz. Each trial lasted 90 seconds, with the kinematic and kinetic data recorded during the last 30 seconds of the trial. All kinematic and kinetic data were acquired via Cortex motion capture software. The work presented here estimated the CoM position as the centroid of the data from the anterior superior iliac spine and posterior superior iliac spine markers [68].

A gait cycle is defined here by two successive touchdowns of a particular foot. This results in a two-step gait cycle, initiated at the first double support (DS) phase [9], with two phases of both legs in stance (DS) and two phases of single leg support (single support, SS). Touchdown and liftoff were defined by the measured vertical GRF exceeding and falling below one percent bodyweight, respectively, to mitigate false positive contact events due to noise in the force data. This threshold ranged between 5N-10N based on subject weight, which lies within the threshold range of 5N-20N used in literature [69]. An average CoM and GRF trajectory for a gait cycle was calculated from a minimum of 12 gait cycles for a given subject-trial pair, with the overall duration of each cycle rescaled based on the average gait duration across all gait cycles. A Fourier series was then fit to the average vertical CoM data using the first eight harmonics. For the fore/aft CoM data, which is in the horizontal direction that the subject is walking, a degree one polynomial was added to the eight harmonics to account for forward progress. The analytical fits were used to interpolate values during the optimization. All handling of human data was completed using custom code in Matlab R2020b, available on GitHub.

### Dynamics formulation

This work focuses on optimizing two specific SLIP-based models: the bipedal SLIP, or B-SLIP model, and the virtual pivot point, or VPP model. The dynamic formulations for these models draw heavy inspiration from previous work in literature [8, 28]. One small difference in this presentation is the measurement of the leg and torso angles about the vertical axis parallel to gravity. This axis definition changes some of the trigonometric conventions for the dynamics, but does not change the underlying physics.

**Fig 2:**
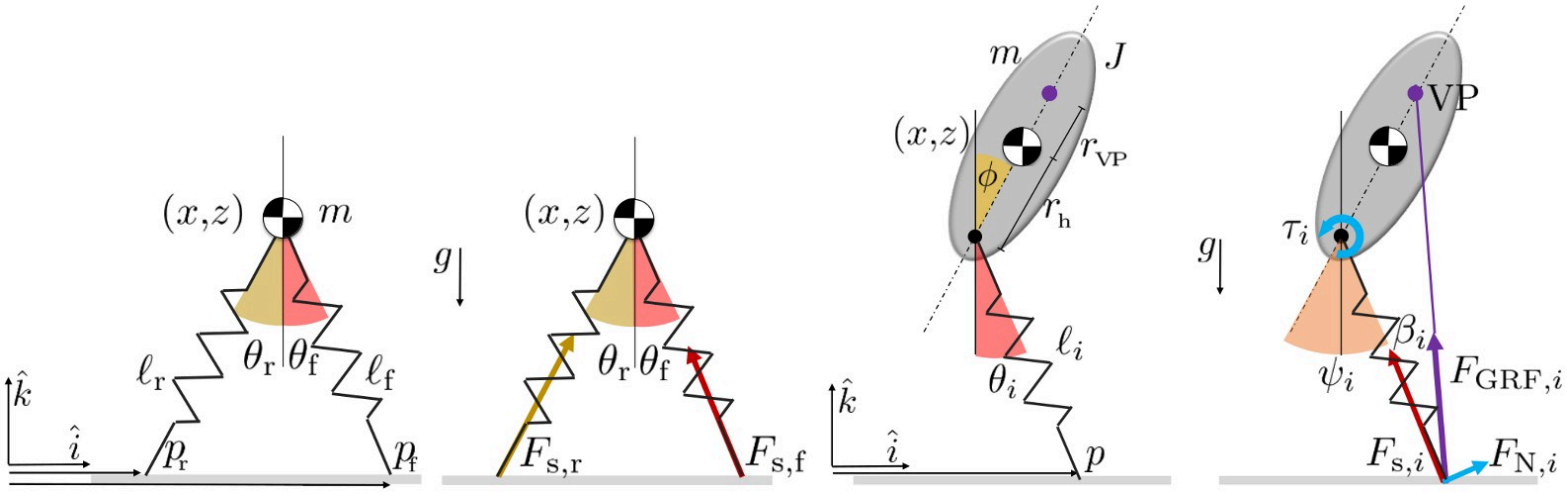
Labeled depictions of the B-SLIP and VPP models, respectively. Note that the VPP model also features two massless spring legs, however the depiction only shows one for a cleaner presentation.

In the B-SLIP model, a point mass *m* is balanced at the hip on massless Hookean springs with spring coefficient *k* and nominal length *ℓ*_0_, as depicted on the left in Fig 2. The compression of the leg generates a spring force *F*_s_ acting along the leg and through the point mass. This force is the total GRF *F*_GRF_ for the B-SLIP.

In the VPP model, the point mass is replaced with a rigid-body trunk that has a moment of inertia *J*, as depicted on the right in Fig 2. The CoM is offset from the hip by a distance *r*_h_. To regulate trunk orientation *ϕ*, a hip torque *τ* is applied to generate a reactionary force *F*_N_ normal to the leg. Combining *F*_N_ and *F*_s_ results in *F*_GRF_ whose line of action intersects a virtual point (VP) at distance *r*_VP_ above the CoM. The hip, CoM, and VP all lie along the axis aligned with *ϕ*.

The state variables of both models include the fore/aft and vertical position (*x*, *z*) and velocity ($\dot{x}$, $\dot{z}$) of the CoM, as well as the stiffness coefficients (*k*_r_, *k*_f_), where (·)_r_ and (·)_f_ denotes rear and front leg, respectively. The VPP model also tracks the orientation and angular velocity (*ϕ*, $\dot{\phi }$) of the trunk. The stiffness coefficients are considered as states such that their rates of change ${\dot{k}}_{r}$ and ${\dot{k}}_{f}$ are the control variables that can be actively modified at each instant. This formulation effectively prevents discrete jumps in leg stiffness between time instances, resulting in smoother GRF profiles. The state vector is then $s={\left[\begin{array}{cccccc} x\;\;\;\;\; & z\;\;\;\;\; & \dot{x}\;\;\;\;\; & \dot{z}\;\;\;\;\; & k_{r}\;\;\;\;\; & k_{f} \end{array}\right]}^{⊤}$ and $s={\left[\begin{array}{cccccccc} x\;\;\;\;\; & z\;\;\;\;\; & \phi \;\;\;\;\; & \dot{x}\;\;\;\;\; & \dot{z}\;\;\;\;\; & \dot{\phi }\;\;\;\;\; & k_{r}\;\;\;\;\; & k_{f} \end{array}\right]}^{⊤}$ for the B-SLIP and VPP model, respectively.

As previously noted in the Introduction, walking involves the coordinated transfer of weight between legs. This coordination translates to instances of SS where forces only act through one leg, and instances of DS where multiple forces are present within the system. In other words, the B-SLIP and VPP models are defined as a hybrid system, with different dynamics between SS and DS. The spring force for a given leg is calculated as

$$\begin{array}{c} F_{s,i}=k_{i}\left(\ell_{0}-\ell_{i}\right) \end{array}$$ (1)

where *ℓ*_*i*_ is the current leg length and *i* ∈ {r, f}. Leg length is geometrically calculated based on foot position *p*_*i*_ and hip position. The hip position coincides with the CoM position for the B-SLIP model, and is found for the VPP using

$$\begin{array}{c} \begin{array}{c} x_{h}=x-r_{h}\;\text{sin}\left(\phi \right)\;\;\;,\;\;\;z_{h}=z-r_{h}\;\text{cos}\left(\phi \right). \end{array} \end{array}$$ (2)

The B-SLIP leg forces are determined trigonometrically, resulting in

$$\begin{array}{c} \begin{array}{ccccc} F_{x,i}=F_{s,i}\;\text{sin}\left(\theta_{i}\right)=F_{s,i}\frac{x_{h}-p_{i}}{\ell_{i}} & & , & & F_{z,i}=F_{s,i}\;\text{cos}\left(\theta_{i}\right)=F_{s,i}\frac{z_{h}}{\ell_{i}}, \end{array} \end{array}$$ (3)

where $F_{x,i}$ and $F_{z,i}$ represent the horizontal and vertical components of the leg force, respectively. Accounting for gravity *g* in the vertical direction, the B-SLIP dynamics are

$$\begin{array}{c} \frac{d}{dt}[\begin{array}{c} \dot{x}\\ \dot{z}\\ k_{r}\\ k_{f} \end{array}]=[\begin{array}{c} \frac{1}{m}\sum_{i}F_{x,i}\\ \frac{1}{m}\sum_{i}F_{z,i}-g\\ {\dot{k}}_{r}\\ {\dot{k}}_{f} \end{array}] \end{array}$$ (4)

where *i* indexes over one leg during SS and both legs in DS to account for the hybrid dynamics.

The VPP model dynamics require a few more calculations due to the inclusion of the rigid trunk. By defining the angle between the leg and the trunk axis as *ψ*_*i*_ = *θ*_*i*_ + *ϕ*, the desired hip torque to redirect forces to the VP is found using

$$\begin{array}{c} \begin{array}{ccccc} \tau_{i}=F_{s,i}\ell_{i}\;\text{tan}\left(\beta_{i}\right) & & , & & \;\text{tan}\left(\beta_{i}\right)=\frac{\left(r_{h}+r_{\text{VP}}\right)\;\text{sin}\left(\psi_{i}\right)}{\ell_{i}+\left(r_{h}+r_{\text{VP}}\right)\;\text{cos}\left(\psi_{i}\right)}. \end{array} \end{array}$$ (5)

A full derivation based on geometric analysis is given in S1 Appendix. The spring force still acts along the leg while the reactionary force from the hip torque acts normal to the leg. Therefore, the VPP fore/aft and vertical forces are determined trigonometrically, resulting in

$$\begin{array}{c} \begin{array}{cc} F_{x,i} & =F_{s,i}\;\text{sin}\left(\theta_{i}\right)-\frac{\tau_{i}}{\ell_{i}}\;\text{cos}\left(\theta_{i}\right)=F_{s,i}\frac{x_{h}-p_{i}}{\ell_{i}}-\tau_{i}\frac{z_{h}}{\ell_{i}^{2}}\\ F_{z,i} & =F_{s,i}\;\text{cos}\left(\theta_{i}\right)+\frac{\tau_{i}}{\ell_{i}}\;\text{sin}\left(\theta_{i}\right)=F_{s,i}\frac{z_{h}}{\ell_{i}}-\tau_{i}\frac{x_{h}-p_{i}}{\ell_{i}^{2}}. \end{array} \end{array}$$ (6)

In total, the VPP model dynamics are then given by

$$\begin{array}{c} \frac{d}{dt}[\begin{array}{c} \dot{x}\\ \dot{z}\\ \dot{\phi }\\ k_{r}\\ k_{f} \end{array}]=[\begin{array}{c} \frac{1}{m}\sum_{i}F_{x,i}\\ \frac{1}{m}\sum_{i}F_{z,i}-g\\ \frac{1}{J}\sum_{i}\left(\tau_{i}-F_{x,i}r_{h}\;\text{cos}\left(\phi \right)+F_{z,i}r_{h}\;\text{sin}\left(\phi \right)\right)\\ {\dot{k}}_{r}\\ {\dot{k}}_{f} \end{array}]. \end{array}$$ (7)

### Optimization formulation

An optimization framework was designed to determine the variables of the template model that best fit its CoM trajectory to walking data. This framework used trajectory optimization, a process of optimizing control variables of a dynamic system relative to some cost function and constraints [70]. The primary goals of the optimization formulation were to a) best fit the template models to human CoM walking data while b) maintaining a highly generalized formulation to mitigate solution overshaping. The optimization formulation was implemented in Matlab R2020b leveraging the CasADi and IPOPT frameworks [71, 72].

A multi-shooting method with Runge-Kutta 4th-order numerical integration and a four-point Gauss-Radau collocation method were considered for the transcription [70]. Collocation was used due to its generally faster convergence and comparable results to multi-shooting. Each phase of the gait cycle was discretized into *N* = 25 finite elements with *M* = 4 collocation points per finite element. The optimization was run over a four-phase gait cycle (4*NM* total points) with respect to the state variables $S=[\begin{array}{cccc} s_{1}\;\;\;\;\; & s_{2}\;\;\;\;\; & \ldots \;\;\;\;\; & s_{4NM} \end{array}]$, and non-state variables $U=[\begin{array}{cccc} u_{1}\;\;\;\;\; & u_{2}\;\;\;\;\; & \ldots \;\;\;\;\; & u_{4NM} \end{array}]$. Since the experimental data was defined with the gait cycle starting at heel strike to initiate the DS phase, the gait cycle for the template model dynamics was also defined to start at heel strike of the first DS phase. This effectively ensures that the experimental and model trajectories are aligned at the start of the gait cycle, similar to as in [9, 35, 36]. The optimization formulation is

$$\begin{array}{ll} \underset{S,U}{\text{min}} & \sum_{j=1}^{4NM}w_{j}\|y_{j}-y_{\text{hum},j}{\|}^{2}\\ \text{s}.\text{t}. & h_{\text{col}}(S,U)=0,\\ & g(S,U)\geq 0,\\ & W_{\text{L}}\leq g_{\text{b}}(S,U)\leq W_{\text{U}} \end{array}$$ (8)

where **y** = [*x z*]^⊤^ and **y**_hum_ = [*x*_hum_ *z*_hum_]^⊤^ vary at each time instant, *w*_*j*_ is the weight factor based on the collocation point, **h**_col_ are equality and collocation constraints, **g** are inequality constraints, and **g**_b_ is used to set bounds on the variables. The **h**_col_ constraints align the endpoints of each element/collocation point to enforce a continuous trajectory and consistent transitions between phases that respect the dynamics (e.g., foot position of support leg at the start of SS must match the foot position of the lead leg at the end of DS). The **g** constraints ensure that variables maintain expected behaviors (e.g., CoM vertical velocity is negative at the start of DS). The **g**_b_ bounds ensure parameters, such as CoM velocity and gait phase durations, stay within a neighborhood feasible for human walking.

Further details are provided for the constraints as follows. Leg stiffness was bounded between 5 and 50 kN/m, which is representative of the range of minimum and maximum stiffness solutions reported for both model simulations and experimental human data [8, 9, 19, 27, 57, 73]. Note that the model simulations tend to favor higher leg stiffness (20+ kN/m) while experimental observations were reported at lower stiffnesses (∼8–15 kN/m). The rate of change of leg stiffness was bound between -100 kN/m/sec and 100 kN/m/sec. Using the average stride times and leg stiffness values reported in [64], we estimated that the average maximum stiffness rate of change was ∼40 kN/m/sec, occurring between the terminal stance (∼9.5 kN/m) and pre-swing phase (∼5.5 kN/m) over ∼0.1 sec. We used 100 kN/m/sec for our bounds since 40 kN/m/sec was an average rate of change, but instantaneous rates of change are most likely larger, and we want optimality to dictate the outcome, rather than the bounds themselves. With these choices, the bounds are sufficiently sized so that they do not impose active constraints upon convergence (i.e., they affect intermediate iterates, but not final results). The objective is simply a running sum of the squared residuals between the optimized and experimental fore/aft and vertical positions of the CoM.

The non-state variables **u** that are optimized consist of variables chosen once per gait phase as well as some that are chosen continuously across each gait phase. For DS and SS, each gait phase optimizes a variable for its time duration *t*_f_ and the starting position(s) *p*_*i*_ of each stance foot (enforcing no foot slip). The rate of change in spring stiffness ${\dot{k}}_{i}$ is chosen independently at each collocation point. For the DS phase, the touchdown angle *θ*_TD_ of the front leg is optimized as an initial condition for the phase. The nominal leg length *ℓ*_0_ is the same across all phases.

The manually chosen parameters are provided in Table 1. Except for gravity, these parameters are specific to the VPP model. The mass for each optimization was set based on the subject mass reported in [67]. The values for *r*_h_ and *J* followed those used in [25, 26, 28]. The values for *r*_VP_ were chosen individually for SS and DS, with DS set to zero based on experimental work that suggested a VP exists only during the SS phase of walking [29]. The authors note that this choice allows for discrete jumps in model hip torque, which is not consistent with human-like behavior. However, the small relative magnitude in force contribution from the hip torque (<5%) mitigates its impact on vertical GRF profile continuity. The value for *r*_VP_ during SS was chosen as a middle-of-the-road between [28, 29]. Several subjects with different weights also had trials optimized at *J* = 6.41 kg m^2^ to check that the choice of rotational inertia did not significantly impact optimization results. The results in S8 Table confirm this. Likewise, to check if choice of distances between hip, CoM, and virtual point impacted stiffness profiles, several subjects with different heights also had trials optimized at *r*_h_ = 0.05*m* and *r*_VP_ = 0.45*m* based on ranges from experimental data [29, 74]. Note that the results from varying *r*_h_ and *r*_VP_, provided in S20 and S21 Figs, suggests that stiffness trends and magnitudes would not be drastically impacted by choice of *r*_h_ and *r*_VP_. This finding does not contradict work in [75] that suggested the choice of magnitude for *r*_h_ impacts effective leg stiffness, because the work herein focuses on ranges applicable for human walking (*r*_h_ ∼ 0 − 0.15m) [74, 76] where the choice of magnitude does not significantly impact stiffness, as opposed to across species and scale.

**Table 1:** Heuristically set template model parameters.

Parameter	Definition	Preset Value
*r*_h_	Distance between CoM and Hip	0.1 m
[DS SS] *r*_VP_	Distance between CoM and VP	[0 0.25]m
*J*	moment of inertia	4.58 kg m^2^
*g*	gravity constant	9.81 m/s^2^

The nonlinear model dynamics prevent global optimum guarantees. To help mitigate convergence to unwanted local optima, initial guesses for the CoM position and velocity, the gait phase durations, and the nominal leg length used data from the walking experiment being optimized. The initial guess for the leg stiffness at touchdown was set to 20 kN/m based on stable gait solutions from [8, 9], while the initial guess for the rate of change in leg stiffness was set to 0 kN/ms. The optimization was run until at least Acceptable status or 500 iterations, whichever occurred first. If the resulting objective cost exceeded a value of 10^−4^, a trial may go through up to four further optimization attempts. Each subsequent attempt was seeded by the optimization result of the previous optimization attempt.

Three quantitative metrics were used to assess model matching of human data. The root mean squared error (RMSE) for CoM tracking, *ϵ*_C_, directly correlates to the objective function of the optimization. This value is non-dimensionalized by the measured subject leg length. The RMSE for vertical GRF matching, *ϵ*_G_, measures salient characteristic retention not explicitly considered in the optimization. This value is non-dimensionalized by the measured subject bodyweight. The RMSE of phase duration matching, $\epsilon_{t_{f}}$, provides a second measure of salient characteristic retention. This value has units of seconds.

The metrics were determined for each data trial and grouped by trial speed. Trials that did not reach an optimal solution were removed from the analysis. The average and standard deviation for each metric within a trial speed were calculated for each model variation. To remove outlier trials that potentially skew results, trials where *ϵ*_C_ exceeded twice the standard deviation within a trial speed were removed. Each remaining trial within a trial speed then went through a ‘remove and compare’ process. The subject-trial pair was pulled out to recalculate the average and standard deviation for the remaining trials of the trial speed. If *ϵ*_C_ for the pulled subject-trial pair exceeded twice the standard deviation, the subject-trial pair was removed from the analysis.

**Fig 3:**
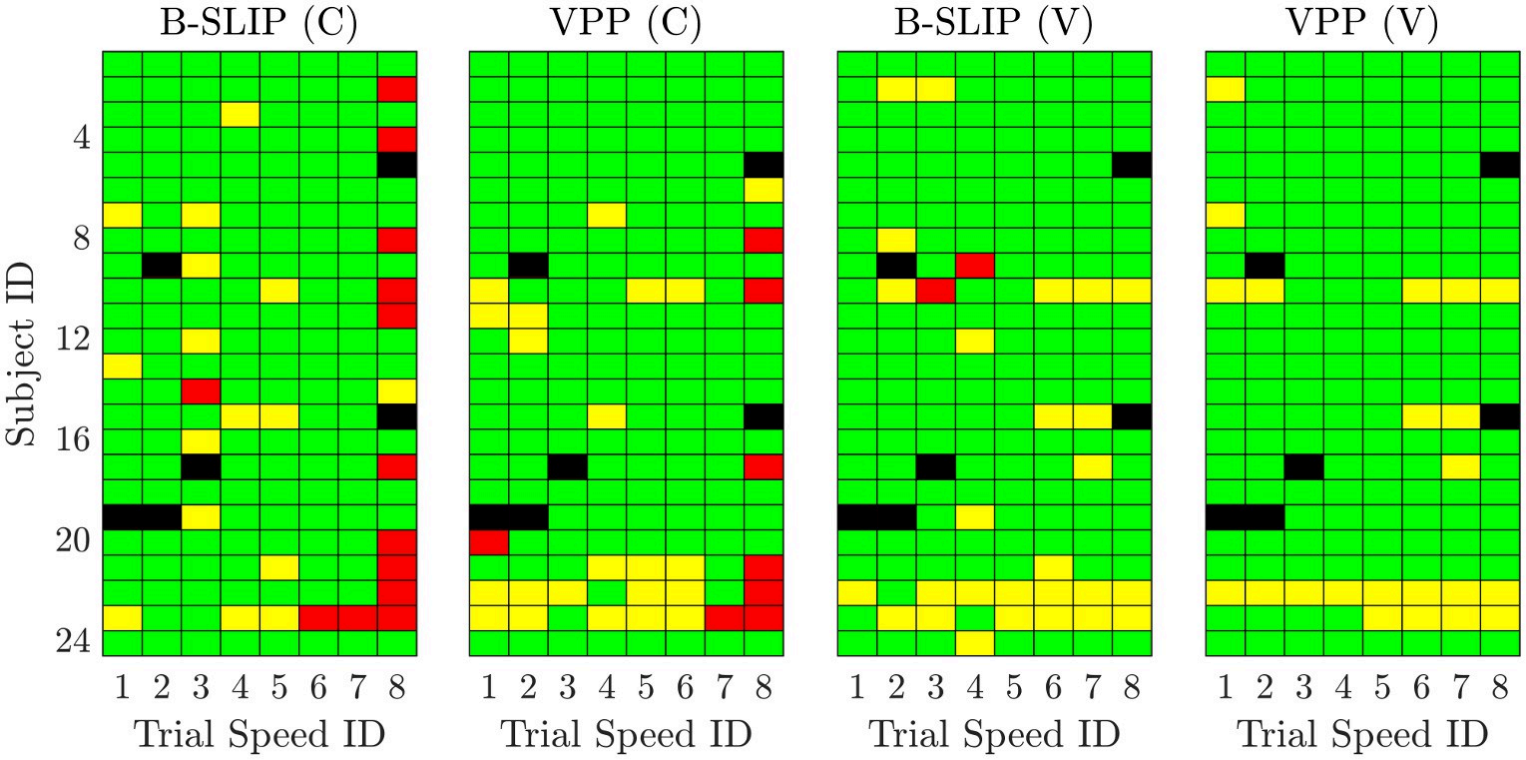
Subject-trial pair inclusion map for metric analysis. Green trials were included, while Yellow trials were removed as outliers. Red trials did not find an optimal solution, and black trials denote trials where the human data could not be processed properly. Trial IDs correspond to percentage of PWS (1: 40%, 2: 55%, 3: 70%, 4: 85%, 5: 100%, 6: 115%, 7: 130%, 8: 145%).

The metric results between model variations were checked for statistical significance. Statistical analysis was conducted for four comparisons: one for each model type (B-SLIP or VPP) to compare stiffness types (constant vs. varying), and one for each stiffness type (constant or varying) to compare model types (B-SLIP vs. VPP). For each of these four comparisons, all-speeds and speed-specific statistical tests were carried out by using a paired, two-sided Wilcoxon signed rank test and a two-sample F-test. The averages and standard deviations for each metric were calculated based on the subject-trial pairs that remained after the outlier removal process noted above. As noted above, some subject-trial pairs were marked as outliers (Fig 3), with each model variation having a different number of subject-trial pairs remaining. This was accounted for when comparing model type variations by only including the data from subjects that were not eliminated from *either* model variation in the statistical analyses. Table 2 lists the number of subject-trial pairs included based on trial speed and model comparison. For both sets of tests, the Wilcoxon signed rank test was used for testing the statistical significance of the subject-to-subject reduction in error. The F-test was used for testing the statistical significance of the change in variance. Unless noted otherwise, results where the p-value is below 0.05 are considered statistically significant. Statistically significant results with p<0.05 are denoted by (*), p<0.005 are denoted by (**), and p<0.0005 are denoted by (***).

**Table 2:** Number of subject-trial pairs included in Wilcoxon statistical analysis.

Trial Speed:	40%	55%	70%	85%	100%	115%	130%	145%	All Trials
B-SLIP (C) vs. B-SLIP (V)	19	18	13	16	19	19	19	11	134
VPP (C) vs. VPP (V)	16	17	22	19	19	19	19	15	146
B-SLIP (C) vs. VPP (C)	16	18	16	19	19	20	23	10	141
B-SLIP (V) vs. VPP (V)	19	17	19	19	21	19	19	19	152

## Results

The optimization framework was conducted on data from 24 individuals at 8 different walking speeds for a total of 192 trials. The minimum and maximum walking speeds were 0.39 m/s and 2.23 m/s, respectively. The optimization framework was applied to the B-SLIP and VPP models with both constant leg stiffness and varying leg stiffness for a total of 768 optimized template models. Constant leg stiffness was achieved by enforcing the rate of change in spring stiffness to always be zero, effectively preventing the stiffness value from changing throughout the gait cycle. Template models with constant and varying leg stiffness are denoted by (C) and (V), respectively.

Across the template model optimization attempts, 721 of the 768 trials reached a successful optimization (Fig 3). Trials where the subject data could not be properly processed are denoted in black. In total, 6 subject-trial pairs failed processing of the human data. Trials where successful optimization was not achieved (maximum attempts exceeded) are denoted in red trials. Note that the higher rate of failed optimizations for the constant stiffness trials at faster walking speeds is expected and aligns with [8, 9], where achieving stable gaits at faster walking speeds was not typically feasible. Trials denoted in yellow resulted in successful optimization but were identified as outliers and removed (see Sec. Methods). Trials denoted in green resulted in successful optimization and passed the outlier check. Except for trial speed 8 for the B-SLIP model with constant leg stiffness, each template model variation retained at least 16 subjects per trial speed. It is noted here that a trial being removed as an outlier does not necessarily indicate a poor optimization for that subject-trial pair. The reader is referred to S1–S16 Figs, where examples of both poor optimization results (Subj. 22 PWS B-SLIP (V) in S13 Fig) and good optimization results but tight inclusion thresholds (Subj. 22 PWS VPP (V) in S13 Fig) resulted in subject-trial pairs being removed. In successful optimizations, constant stiffness models averaged ∼40 seconds for the first optimization pass, and ∼6 seconds for subsequent optimizations if the objective cost threshold was exceeded. Varying stiffness models averaged ∼30 seconds for the first optimization pass, and ∼3 seconds for subsequent optimizations.

### Center of mass analysis

**Fig 4:**
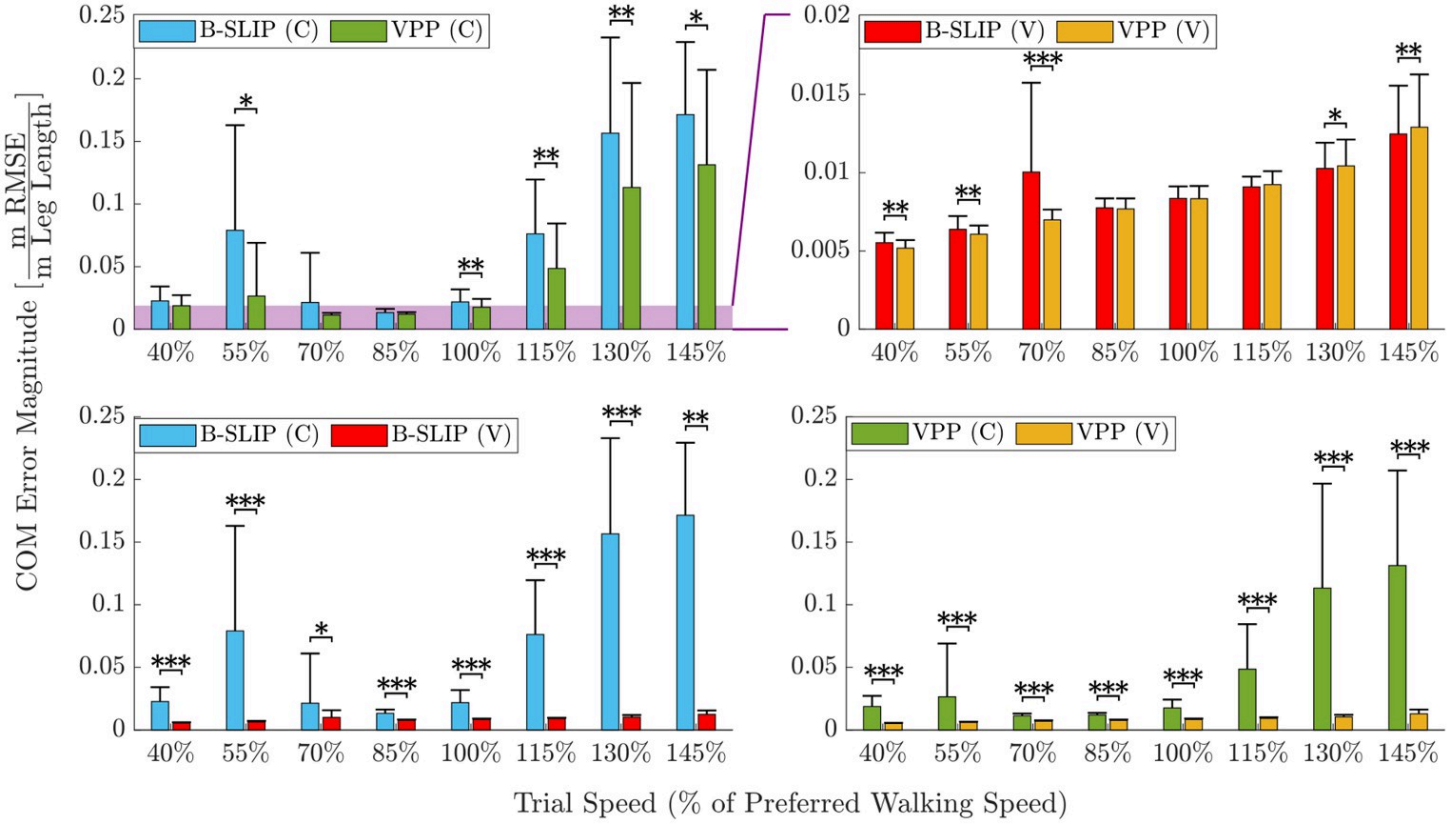
Average and standard deviation *ϵ*_C_ by trial speed. Top compares model type and bottom compares stiffness type (constant-C, varying-V). The purple area gives axis comparison between the top figures. Asterisks denote statistically significant error differences (*p<0.05, **p<0.005, ***p<0.0005). P-values are provided in S1 and S4 Tables.

Average CoM tracking values with corresponding standard deviations per trial speed are provided in Table 3 and illustrated in Fig 4. The top figures compare error results between model types, while the bottom figures compare error results between stiffness types. Comparing stiffness types, optimizing with varying leg stiffness reduced average error by 87.03%±30.02% (avg. ± std. dev.) (***) for the B-SLIP model and 82.01%±21.53% (***) for the VPP model. Optimizing with varying leg stiffness reduced standard deviation by 95.78% (***) for the B-SLIP model and 95.68% (***) for the VPP model. All of these results included a comparison using between 134–152 trials (Table 2).

**Table 3:** Center of mass tracking error values *ϵ*_C_ (average±standard deviation).

Trial Speed:	40%	55%	70%	85%	100%	115%	130%	145%	All Trials
B-SLIP (C)	0.0227 ±0.0115	0.0791 ±0.0839	0.0214 ±0.0397	0.0133 ±0.0030	0.0218 ±0.0100	0.0762 ±0.0433	0.1566 ±0.0764	0.1714 ±0.0579	0.0621 ±0.0745
B-SLIP (V)	0.0055 ±0.0006	0.0064 ±0.0008	0.0100 ±0.0057	0.0078 ±0.0006	0.0084 ±0.0008	0.0091 ±0.0007	0.0103 ±0.0016	0.0125 ±0.0031	0.0087 ±0.0031
VPP (C)	0.0118 ±0.0085	0.0265 ±0.0425	0.0114 ±0.0018	0.0120 ±0.0016	0.0176 ±0.0067	0.0485 ±0.0359	0.1133 ±0.0834	0.1313 ±0.0758	0.0462 ±0.0617
VPP (V)	0.0052 ±0.0005	0.0061 ±0.0006	0.0070 ±0.0007	0.0077 ±0.0007	0.0083 ±0.0008	0.0092 ±0.0009	0.0104 ±0.0017	0.0129 ±0.0034	0.0083 ±0.0027

Comparing model types, optimizing the VPP model reduced average error by 31.03%±74.32% (***) for constant stiffness, and by 4.34%±11.53% (p = 0.1075) for varying stiffness compared to the B-SLIP model. While the p-value for the comparison of the constant stiffness models is statistically significant, the large standard deviation with respect to the average suggests that caution should be taken to avoid over-interpretation. Optimizing the VPP model reduced the standard deviation by 17.21% (*) for constant stiffness and by 15.29% (*) for varying stiffness, both as compared to the B-SLIP model. S1 and S4 Tables provide statistical significance by trial speed.

**Fig 5:**
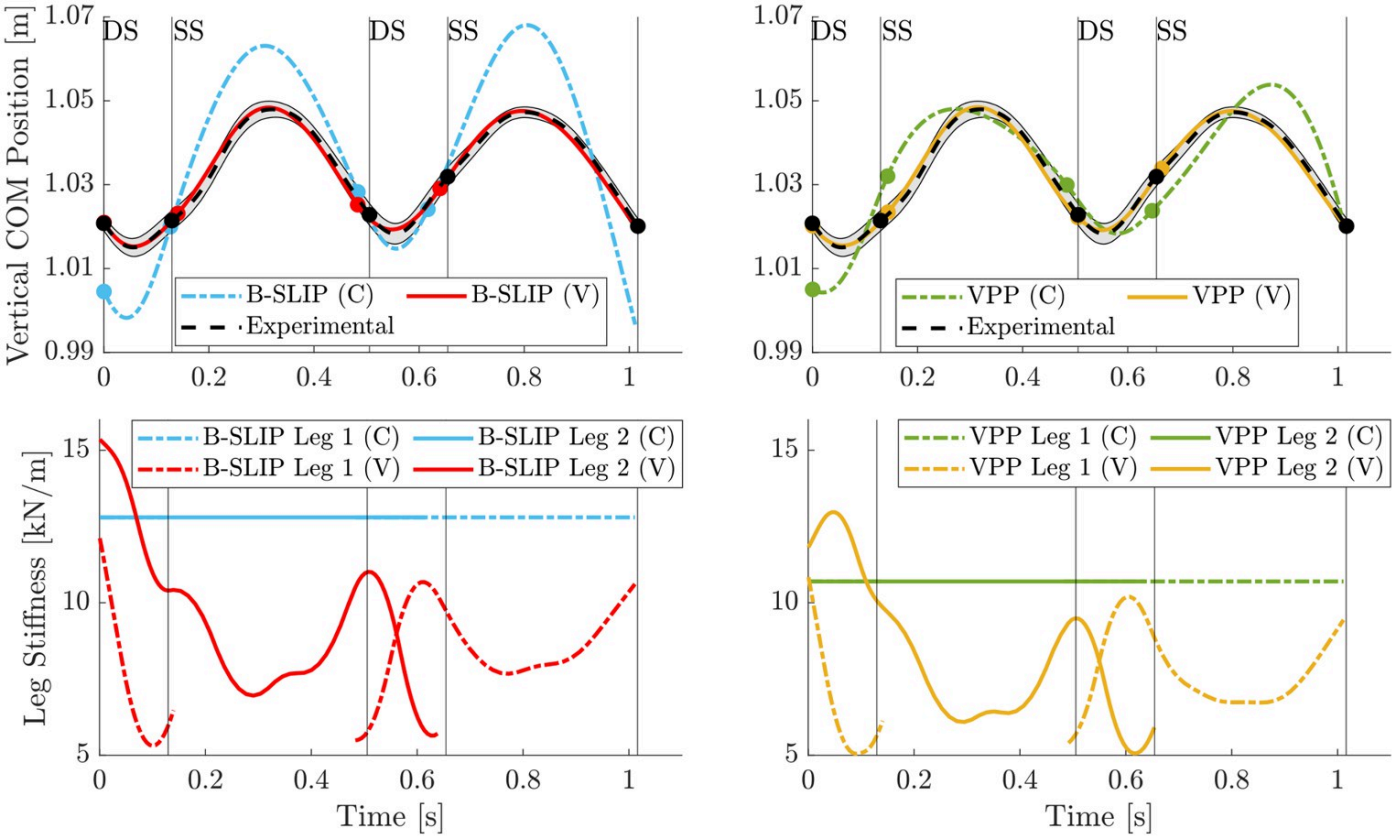
Vertical CoM and leg stiffness profile results for Subject 4 at PWS. B-SLIP and VPP model variations in left and right figures, respectively. Markers and vertical lines denote gait events. Shaded region denotes standard deviation of average gait cycle.

The vertical CoM trajectories and stiffness profiles for each model variation for Subject 4 walking at their PWS of 1.3 m/s (Trial 5) is provided in Fig 5. The left figure compares B-SLIP model variations, while the right figure compares VPP model variations. In both model types, the varying leg stiffness variations track the CoM data within the standard deviation throughout the gait cycle. The models with varying stiffness spanned between 5 and 16 kN/m for the B-SLIP model, and between 5 and 14 kN/m for the VPP model. The models where the stiffness was held constant resulted in a stiffness value above the average stiffness of the varying stiffness models, 12.8 kN/m and 10.7 kN/m for the B-SLIP and VPP, respectively. Additional subject-trial pair results are available in S1, S3, S5, S7, S9, S11, S13 and S15 Figs. The average and peak stiffness rate of change during the pre-swing, or second DS, phase of the gait cycle is provided in Table 4 for four subjects who have similar preferred walking speeds as the walking speed reported in [64]. The average stiffness rate of change values are within a factor of two of the estimated rate of change in [64], and the peak values are well within the bounds set in the optimization framework.

**Table 4:** Stiffness rate of change $\dot{k}$ during pre-swing phase.

Subject	Walking Speed (m/s)	Average $\dot{k}$ (kN/m/sec)	Peak $\dot{k}$ (kN/m/sec)
Subject 07	1.10	35.92	81.83
Subject 09	1.17	25.65	56.27
Subject 12	1.05	21.52	45.57
Subject 16	1.10	23.37	53.91
Wang et. al [64]	1.11	∼40	N/A

### Ground reaction force analysis

**Fig 6:**
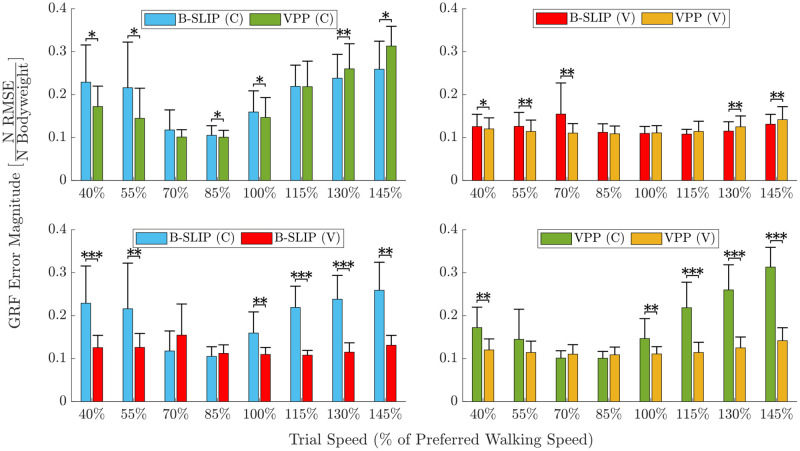
Average and standard deviation *ϵ*_G_ by trial speed. Top compares model type and bottom compares stiffness type (constant-C, varying-V). Asterisks denote statistically significant error differences (*p<0.05, **p<0.005, ***p<0.0005). P-values are provided in S2 and S5 Tables.

Average GRF matching metric values with corresponding standard deviations per trial speed are provided in Table 5 and illustrated in Fig 6. The top figures compare error results between model types, while the bottom figures compare error results between stiffness types. Comparing stiffness types, optimizing with varying leg stiffness reduced average error by 36.07%±39.58% (***) for the B-SLIP model and 34.27%±32.16% (***) for the VPP model. Optimizing with varying leg stiffness reduced standard deviation by 57.24% (***) for the B-SLIP model and 69.95% (***) for the VPP model.

**Table 5:** Ground reaction force error values *ϵ*_G_ (average±standard deviation).

Trial Speed:	40%	55%	70%	85%	100%	115%	130%	145%	All Trials
B-SLIP (C)	0.2290 ±0.0866	0.2160 ±0.1062	0.1177 ±0.0466	0.1052 ±0.0224	0.1595 ±0.0492	0.2192 ±0.0494	0.2383 ±0.0555	0.2591 ±0.0653	0.1918 ±0.0826
B-SLIP (V)	0.1256 ±0.0286	0.1261 ±0.0325	0.1546 ±0.0725	0.1122 ±0.0198	0.1098 ±0.0162	0.1080 ±0.0111	0.1150 ±0.0218	0.1312 ±0.0229	0.1226 ±0.0353
VPP (C)	0.1723 ±0.0474	0.1448 ±0.0701	0.1012 ±0.0171	0.1008 ±0.0160	0.1467 ±0.0464	0.2184 ±0.0594	0.2600 ±0.0584	0.3131 ±0.0460	0.1790 ±0.0841
VPP (V)	0.1202 ±0.0257	0.1143 ±0.0264	0.1104 ±0.0221	0.1088 ±0.0180	0.1113 ±0.0166	0.1142 ±0.0237	0.1250 ±0.0252	0.1417 ±0.0302	0.1177 ±0.0253

Comparing model types, optimizing the VPP model reduced average error by 6.63%±26.24% (p = 0.2822) for constant stiffness and 4.00%±14.08% (p = 0.2283) for varying stiffness compared to the B-SLIP model. Optimizing the VPP model increased standard deviation by 1.81% (p = 0.8232) for constant stiffness and decreased it by 28.46% (***) for varying stiffness compared to the B-SLIP model. S2 and S5 Tables provide statistical significance by trial speed.

**Fig 7:**
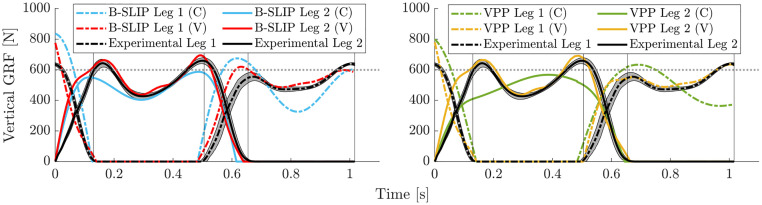
Vertical GRF results for optimization of Subject 4 at PWS. B-SLIP and VPP model variations in left and right figures, respectively. Shaded region denotes standard deviation of subject’s average vertical GRF profile.

The resulting vertical GRF profiles for each template model variation for Subject 4 walking at their PWS of 1.3 m/s (Trial 5) are provided in Fig 7. The left figure compares B-SLIP model variations, while the right figure compares VPP model variations. In both model types, the models with varying stiffness visually track the subject data better and more consistently. Furthermore, the varying stiffness models visually track the asymmetry of the second M-peak profile better than the constant stiffness models. Notable error outside of the standard deviation occurs during the initial peak rise upon foot touchdown for the varying stiffness models. Additional subject-trial pair results are available in S2, S4, S6, S8, S10, S12, S14, and S16 Figs.

### Gait event timing analysis

**Fig 8:**
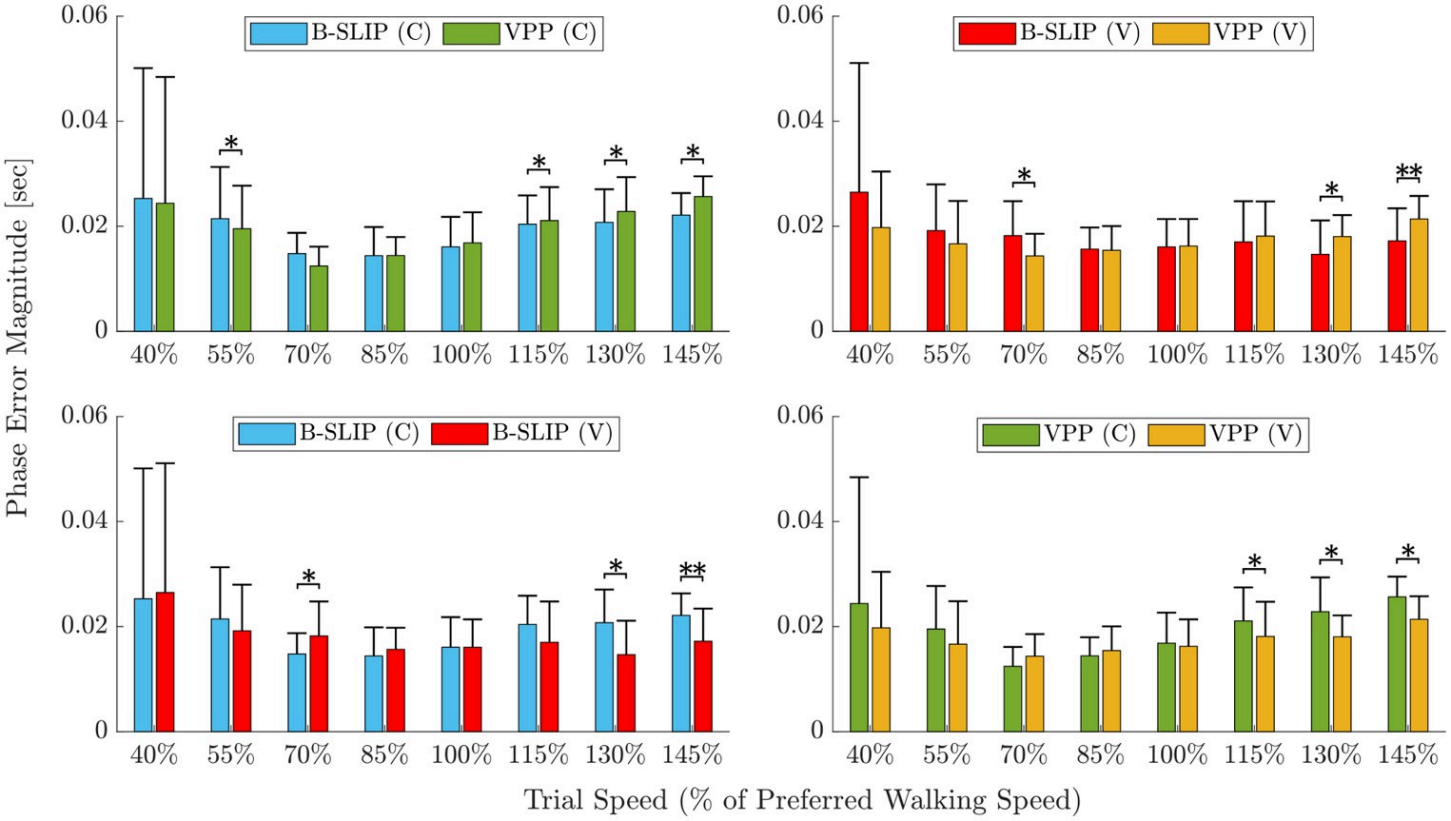
Average and standard deviation $\epsilon_{t_{f}}$ by trial speed. Top compares model type and bottom compares stiffness type (constant-C, varying-V). Asterisks denote statistically significant error differences (*p<0.05, **p<0.005, ***p<0.0005). P-values are provided in S3 and S6 Tables.

Average values for gait event matching with corresponding standard deviations per trial speed are provided in Table 6 and illustrated in Fig 8. The top figures compare error results between model types, while the bottom figures compare error results between stiffness types. Comparing stiffness types, optimizing with varying leg stiffness reduced average error by 6.21%±43.33% (*) for the B-SLIP model and 10.30%±41.71% (p = 0.0636) for the VPP model. Optimizing with varying leg stiffness increased standard deviation by 3.38% (p = 0.6788) for the B-SLIP model, and reduced standard deviation by 37.81% (***) for the VPP model. Similar to the comparison of constant stiffness models with CoM tracking performance, the large standard deviations with respect to the average reductions in error suggest that improvement in performance is subject-trial specific, and caution should be taken to avoid over-interpretation.

**Table 6:** Gait event timings error values $\epsilon_{t_{f}}$ (average±standard deviation).

Trial Speed:	40%	55%	70%	85%	100%	115%	130%	145%	All Trials
B-SLIP (C)	0.0253 ±0.0248	0.0214 ±0.0099	0.0148 ±0.0040	0.0144 ±0.0055	0.0161 ±0.0057	0.0204 ±0.0055	0.0207 ±0.0063	0.0221 ±0.0042	0.0194 ±0.0110
B-SLIP (V)	0.0265 ±0.0246	0.0192 ±0.0088	0.0182 ±0.0066	0.0157 ±0.0041	0.0161 ±0.0053	0.0170 ±0.0078	0.0147 ±0.0065	0.0172 ±0.0062	0.0182 ±0.0114
VPP (C)	0.0244 ±0.0241	0.0195 ±0.0082	0.0124 ±0.0037	0.0144 ±0.0035	0.0168 ±0.0058	0.0211 ±0.0064	0.0228 ±0.0065	0.0257 ±0.0038	0.0194 ±0.0105
VPP (V)	0.0198 ±0.0107	0.0167 ±0.0082	0.0143 ±0.0042	0.0154 ±0.0046	0.0162 ±0.0050	0.0181 ±0.0066	0.0180 ±0.0041	0.0214 ±0.0044	0.0174 ±0.0065

Comparing model types, the difference in error between varying and constant stiffness for the VPP model was ≈0% (p = 0.2135), while varying stiffness reduced error by 4.43%±54.48% (p = 0.3616) for the B-SLIP model. Optimizing the VPP model reduced standard deviation by 4.69% (p = 0.5496) for constant stiffness and 42.66% (***) for varying stiffness compared to the B-SLIP model. S3 and S6 Tables provide statistical significance by trial speed.

Visual markers of gait events for each model variation are provided in Fig 5 for Subject 4 walking at their PWS of 1.3 m/s (Trial 5). The vertical lines denote the instances of gait events for the subject data, while the markers denote the instances of gait events for each graphed trajectory. For this trial, the varying stiffness model variations better align with the gait event timings for the subject data than their constant stiffness model counterparts. Other subject-trial pairs provided in the Supplementary demonstrate the trial-specificity of which model/stiffness variation captured gait event timings best.

## Discussion

This work looked to answer three research questions regarding the constant stiffness assumption for SLIP-type models, specifically the B-SLIP and VPP models. The results from applying the optimization framework outlined above to these models demonstrate that allowing the leg stiffness to vary throughout the gait cycle improves CoM trajectory tracking with statistical significance across subjects and walking speeds, as proposed in H1. Models with varying stiffness also showed statistically significant improvement in GRF profile matching, highlighting the retention of salient characteristics, as proposed in H2. Improvement in gait event timings was subject-trial pair specific, leaving H3 currently inconclusive.

### CoM tracking

Looking at the outcomes with respect to H1, there was a statistically significant reduction in CoM tracking error and standard deviation when the leg stiffness was allowed to vary throughout the gait cycle. This improvement held for both the B-SLIP and VPP model across individual subjects, and across walking speeds. With over 80% improvement in CoM tracking for both model types, many of the resulting CoM trajectories for the models with varying stiffness lie within a single standard deviation of the experimental trajectory, as illustrated in Fig 5. That is, the improvements achieved by the models with varying stiffness result in CoM trajectories that lie within the observed step-to-step variance of human walking [76, 77], making the methodology relevant to human-centric applications, such as use of target CoM references generated by these varying stiffness models for the control of prostheses and exoskeletons. These results also align with the ability of stiffness variation to address irregularities such as a less sinusoidal vertical CoM trajectory observed at slower walking speeds [67]. Improvement in CoM tracking suggests that encoding the non-conservative actions of leg muscles and joint actuation [55, 78–80] into a one-dimensional parameter can still retain the information previously analyzed through two-dimensional GRF profiles [19, 32, 36] or multi-dimensional joint space [58, 59, 61, 79].

Looking beyond human locomotion, it is hypothesized that the benefits of varying leg stiffness would extend well to other legged animals. SLIP-based models already possess the innate ability to scale across weight and size [12, 13], which does not change by simply allowing the leg stiffness to vary. More specifically, locomotion patterns that do not produce sinusoidal trajectories, such as certain species of lizards [81] or crabs [82], are expected to have the most to gain. This hypothesis is driven by the finding that variable stiffness offered the most critical benefit for matching CoM trajectories during the less sinusoidal evolution observed in humans walking below their preferred speeds [67]. A future direction for this work would investigate how to extend these SLIP-based methods to multi-legged locomotion (four-, six-, eight-) depending on contact sequencing [16].

### GRF matching

Interesting trend differences between constant stiffness models vs. varying stiffness models arise when analyzing the outcomes with respect to H2. For constant stiffness models, the GRF matching error is lowest around 85% PWS, and increases nonlinearly away from this speed. For the varying stiffness models, GRF matching error is near invariant with respect to trial speed. The statistically significant improvement in GRF matching error across speeds suggests that models with varying stiffness better capture GRFs compared to constant stiffness models, and the invariant trend across speeds suggests this improvement is independent of walking speed. This invariance is particularly notable for speeds lower than PWS, where the GRF profiles shifts from an M-shape towards a single peak [83, 84].

This result is particularly notable because the use of spring-like dynamics was investigated from the perspective of CoM progression throughout the gait cycle, as opposed to directly from the GRF as done in the literature [19, 21, 35–37]. The work here demonstrates that both CoM kinematics and GRF profiles are able to be accurately captured in SLIP-based models with varying stiffness, even if only CoM position is explicitly considered in the optimization. The retention of GRF information without being explicitly considered in the optimization framework further supports the extensive literature surrounding the spring-like behavior of legged locomotion [7, 8, 13, 85, 86]. As previously noted, this result is achieved via focus on only the one-dimensional leg stiffness variations, rather than via considering 2-D GRFs directly.

Looking beyond human locomotion, the methodology used in this work is hypothesized to handle GRF prediction for other animals well. Many animals exhibit the characteristic M-shape profile for vertical GRFs that are seen in human walking, including quadrupedal animals like giraffes and bears [87, 88], as well as smaller multi-legged animals like caterpillars [89]. As previously highlighted, at slower walking speeds, the M-shape profile starts to resemble more of a single plateau, which was still able to be captured by the varying stiffness models. This suggests that animals who exhibit single-peak GRF profiles like bipedal monkeys [90], or even extreme cases like quadrupeds walking in a tripod gait due to amputation [91] can still be represented by the work presented here.

### Gait event matching

Analyzing the outcomes with respect to H3, improvements in gait event duration matching were observed to be trial specific. For models both with constant stiffness and varying stiffness, matching gait event timings was more accurate closer to a subject’s PWS. The varying stiffness models achieved statistically significant improvement only at faster walking speeds, which aligns with issues for the B-SLIP model to generate walking trajectories as speed increases [8, 9]. Subject-specific gait event timings, such as Subj. 1 and 19 having the same PWS of 1.21 m/s but different gait event timings (S7 Table), may play a role in the improvement being subject-trial pair dependent. This lower improvement may also be partially based on the method of error calculation, which compares each phase duration between the template model and the human data, rather than the timing of the gait events in the overall gait cycle. While all template models are aligned to initiate the gait cycle at the instance of DS, this can lead to double penalizations in the error calculation when one phase duration attempts to correct for timing issues occurring in a previous phase.

It is important to note that the optimization framework and the analysis in this work was based on absolute time since heelstrike at the start of DS, rather than doing the same based on a relative timing measure such as gait percentage or aligning individual gait events [92, 93]. This decision translates back to the underestimation of gait timings by previous SLIP-based models [9]. Since we are interested in the overall evolution of these SLIP-based models for use in applications related to human-robot interactions in rehabilitation, it is important that the overall timing sequence of these models aligns with what’s been observed experimentally for humans. This was a primary motivator to use overall time duration, as opposed to other relative timing measures that one would get when considering gait percentage or dynamic time warping.

It should be noted that the choice of temporal alignment constraints used herein plays a determining factor in the outcome of which model variation (constant or varying) achieved better gait event matching. To illustrate, the optimization for the B-SLIP model was re-run for several subject-trial pairs where a) all constraints related to temporal alignment were removed, and b) where only the constraint pertaining to total gait duration was active. These results are provided in S22 Fig, which demonstrate that the varying stiffness models more consistently outperform constant stiffness models in gait event matching without those constraints active. However, for constant stiffness models, removing the timing constraints resulted in poor quality (locally optimal) solutions that would not be feasible for human walking (see S22 Fig). Therefore, the constraints were kept active for this work to ensure that the comparison of CoM tracking performance between constant and varying stiffness models could be considered on equal footing.

### Stiffness profiles

Outside of the three hypotheses proposed, the resulting profiles of both the constant and varying stiffness models show interesting trends. The varying stiffness profiles resemble some characteristics expected during human walking. Looking at Fig 5, the leg stiffness has higher variation in the DS phase than the SS phase. The larger variation is most likely capturing the work requirement for redirecting the CoM velocity [94] during the collision events, which aligns with the minimal biped model with telescoping actuators from Srinivasan and Ruina [10]. During the SS phases, leg stiffness is reduced, which aligns with when the overestimation of vertical CoM displacement is most prominent in the constant stiffness model [9]. The leg stiffness inflects around halfway through SS, suggesting that the two halves of stance are more accurately represented by separate stiffnesses [95]. Looking at Fig 5, the front leg increases stiffness while the back leg decreases stiffness during the DS phase, mimicking the weight acceptance for CoM velocity redirection and ankle push-off that is critical to human walking [96]. The overall trends of higher stiffness (∼15kN/m) at the beginning of the stance phase for a given leg, with lowering stiffness (∼7–9kN/m) in the middle of stance, ending with lower stiffness (∼5kN/m) near the end of the stance phase for the same leg also align very well with the values reported in [64]. For reference, [64] reported ∼15kN/m in early stance, ∼9–14kN/m in mid-stance, and ∼5kN/m near the end of stance.

Comparing across speeds, leg stiffness profiles appear to decrease in magnitude as speed increases for a given subject (Fig 5, S1 and S3 Figs for Subject 4), (S5, S7 and S9 Figs for Subject 13), (S11, S13 and S15 Figs for Subject 22). This is most notable for the varying stiffness models looking at the average magnitude of the SS phase, whereas the trend is present for the constant stiffness models where the resulting CoM trajectory is qualitatively close to the experimental trajectory (S1 Fig vs S3 Fig as a reference). This negative correlation between leg stiffness and speed aligns with previous findings in the literature for both experimental data [57] and SLIP-based models [9] for walking. However, for running gaits in humans, leg stiffness has been found to be relatively speed invariant [9, 60, 73, 97], which was not studied in this work. Leg stiffness was reported with a positive correlation to speed for walking in [19], however their model included a damper, which introduces dynamic interplay that may influence the resulting stiffness magnitude.

Looking at the range of magnitudes for the leg stiffness profiles, both constant and varying stiffness models resulted in stiffnesses typically between 5–30kN/m. More specifically, the average magnitude of the SS phase for the varying stiffness models typically resided in the 5–20kN/m range, most often <15kN/m. This range aligns well with ranges reported experimentally in the literature (4–10 kN/m [57], 16–35 kN/m [9], 9–22 kN/m [60], 3–15 kN/m [64]).

Furthermore, the average stiffness rate of change for several subjects whose PWS was close to the walking speed reported in [64] was found to be similar (21–35 kN/m/sec vs. ∼40 kN/m/sec) to the estimated average rate of change in [64] during the pre-swing, or second DS, phase. The peak rate of change was well within the 100 kN/m/sec bound set in the optimization framework, meaning the bounds chosen were not a limiting factor to the resulting optimal trajectories. These findings are being compared to estimations from [64] since, to the authors’ knowledge, there is not currently any literature on experimentally obtained stiffness rate of change parameters. However, the outcomes suggest that the optimization is not resulting in trajectories far beyond what may be biomechanically plausible.

For some of the trials (e.g., S13 and S15 Figs), the resulting CoM trajectory for the constant stiffness model may achieve better matching with experimental CoM data if a lower stiffness value is chosen; however, such solutions would poorly match the gait event times. Thus, the optimal solution is governed by the temporal alignment constraints, where the lower stiffness magnitude would result in too large a discrepancy in gait cycle duration. In other words, the constant leg stiffness models are more sensitive to the balance between tracking CoM characteristics and matching human-like durations for different gait phases like SS and DS, compared to varying stiffness models. This sensitivity is highlighted in S22 Fig.

### B-SLIP vs. VPP

Comparing model types for the CoM tracking and GRF matching, the VPP model outperforms the B-SLIP model with statistical significance for speeds below PWS, while the B-SLIP model outperforms the VPP with statistical significance for speeds above PWS. The statistically better performance of the VPP model at lower trial speeds supports the importance of transversal forces and hip torque regulation at slower speeds, while the axial forces along the legs begin to dominate at higher walking speeds [95]. This performance switch suggests that posture regulation in the VPP model plays a more significant role at lower speeds [98] and less of a role at higher speeds, which aligns with findings of reduced hip stiffness and increased hip rest angle as locomotion speed increased [95].

### Limitations

The authors note the following considerations for future investigations that may address limitations of this work. Varying the leg stiffness at each time instant increases the number of model parameters that must be optimized, and may lead to overfitting the data. Future work may seek a further reduced parameterization to still accurately capture human walking characteristics while addressing these concerns. All of the data came from treadmill walking, which does not fully capture the variability in walking speed and the surfaces of overground walking [34]. Similarly, this work was limited to walking gaits on a rigid surface, with each optimization considered over a single averaged gait cycle. However, the authors hypothesize that by extending these methods to multi-step cycles to allow for different terrains and compliant surfaces across steps, varying leg stiffness models will offer similar levels of benefits for reconstructing CoM and GRF profiles compared to constant stiffness models. This hypothesis is motivated by the finding in [34] where it was reported that even though vertical displacement into the ground surface increased as surface stiffness decreased, the total vertical displacement of the CoM of the individual did not change. This discrepancy could plausibly be explained by a varying stiffness model, which could better retain total vertical displacement of the CoM compared to constant stiffness models. The bounds on the rate of change in stiffness used in this work were to maintain smooth stiffness profiles *within a single stride*, but this does not impact the ability of the model to adjust to surface compliance from step to step. Finally, the data used came from individuals with no physical impairments. Pathological walking data may lead to the discovery of how stiffness profiles are impacted by injury and the impact of the constant stiffness assumption on predictive analysis of human walking [64, 74, 99, 100].

### Future work

Looking forward, the extension of this work to simulating the SLIP-based models with the optimized stiffness profiles may lead to insights into how leg length and touchdown angle alone could handle stride-to-stride variations in the CoM trajectory and GRF profiles. Such next steps could improve the accuracy of these models for predictive analysis, which is not otherwise possible if parameterizing ground forces directly.

This work also provides the basis for ongoing research on task-level control for lower-limb prostheses [101]. The predicted GRF profiles determined from the optimized reference trajectory set the desired open loop dynamics and map onto torque commands for the joints. Feedback impedance control is implemented via force corrections to deviations between real-time CoM kinematics and reference CoM trajectories that are consistent with target template dynamics. Testing of this ankle prosthesis control with individuals with transtibial amputation has been completed and is undergoing data analysis, with extensions to knee-ankle prostheses planned in the near future. The end goal will be for the results from this current study to directly guide the framework and ensure that stiffness variation control strategies originally designed for robots [66], are implemented in a manner that is compatible with CoM-level considerations of the human motor control system.

## Conclusions

This paper has analyzed the role of fixed-stiffness assumptions on the ability of compliant-leg models to describe experimentally observed CoM and GRF data. While allowing the leg stiffness to vary in time can only improve the ability to fit CoM trajectories, the findings show that this single time-varying parameter is enough to eliminate the majority of the CoM reconstruction error observed in the fixed-stiffness case, and despite the model only focusing on modulating a single direction for the ground forces. Further, while measured GRF profiles were used to assess the results, the proposed optimization framework does not require them as input, opening the door for this framework to be used for inferring ground force or leg stiffness trajectories to be used on assistive devices in future work.

## Supporting information

S1 Appendix Geometric derivation of Eq (5) for the VPP model. (PDF)

S1 Table Center of mass tracking error Wilcoxon test by trial speed. (PDF)

S2 Table Ground reaction force matching error Wilcoxon test by trial speed. (PDF)

S3 Table Gait event matching error Wilcoxon test by trial speed. (PDF)

S4 Table Center of mass tracking standard deviation F-test by trial speed. (PDF)

S5 Table Ground reaction force matching standard deviation F-test by trial speed. (PDF)

S6 Table Gait event matching standard deviation F-test by trial speed. (PDF)

S7 Table Comparison of gait event timings between various subject trials. (PDF)

S8 Table Effect of mass inertia parameter on optimization results at PWS. (PDF)

S1 Fig Vertical CoM and leg stiffness results for Subject 4 at 70% PWS. B-SLIP and VPP model variations in left and right figures, respectively. Markers and vertical lines denote gait events. Shaded region denotes standard deviation of average gait cycle. All template models achieved optimal solutions and passed the outlier screening for this subject-trial pair.
(PDF)

S2 Fig Vertical GRF results for optimization of Subject 4 at 70% PWS. B-SLIP and VPP model variations in left and right figures, respectively. Shaded region denotes standard deviation of subject’s average vertical GRF profile.
(PDF)

S3 Fig Vertical CoM and leg stiffness results for Subject 4 at 130% PWS. B-SLIP and VPP model variations in left and right figures, respectively. Markers and vertical lines denote gait events. Shaded region denotes standard deviation of average gait cycle. All template models achieved optimal solutions and passed the outlier screening for this subject-trial pair.
(PDF)

S4 Fig Vertical GRF results for optimization of Subject 4 at 130% PWS. B-SLIP and VPP model variations in left and right figures, respectively. Shaded region denotes standard deviation of subject’s average vertical GRF profile.
(PDF)

S5 Fig Vertical CoM and leg stiffness results for Subject 13 at 70% PWS. B-SLIP and VPP model variations in left and right figures, respectively. Markers and vertical lines denote gait events. Shaded region denotes standard deviation of average gait cycle. All template models achieved optimal solutions and passed the outlier screening for this subject-trial pair.
(PDF)

S6 Fig Vertical GRF results for optimization of Subject 13 at 70% PWS. B-SLIP and VPP model variations in left and right figures, respectively. Shaded region denotes standard deviation of subject’s average vertical GRF profile.
(PDF)

S7 Fig Vertical CoM and leg stiffness results for Subject 13 at 100% PWS. B-SLIP and VPP model variations in left and right figures, respectively. Markers and vertical lines denote gait events. Shaded region denotes standard deviation of average gait cycle. All template models achieved optimal solutions and passed the outlier screening for this subject-trial pair.
(PDF)

S8 Fig Vertical GRF results for optimization of Subject 13 at 100% PWS. B-SLIP and VPP model variations in left and right figures, respectively. Shaded region denotes standard deviation of subject’s average vertical GRF profile.
(PDF)

S9 Fig Vertical CoM and leg stiffness results for Subject 13 at 130% PWS. B-SLIP and VPP model variations in left and right figures, respectively. Markers and vertical lines denote gait events. Shaded region denotes standard deviation of average gait cycle. All template models achieved optimal solutions and passed the outlier screening for this subject-trial pair.
(PDF)

S10 Fig Vertical GRF results for optimization of Subject 13 at 130% PWS. B-SLIP and VPP model variations in left and right figures, respectively. Shaded region denotes standard deviation of subject’s average vertical GRF profile.
(PDF)

S11 Fig Vertical CoM and leg stiffness results for Subject 22 at 70% PWS. B-SLIP and VPP model variations in left and right figures, respectively. Markers and vertical lines denote gait events. Shaded region denotes standard deviation of average gait cycle. All template models achieved optimal solutions, but only B-SLIP (C) passed the outlier screening. Note that B-SLIP (V) achieved a poor optimization, yet VPP (V) tracked vertical CoM within a standard deviation the entire gait cycle. This is an example of removal from analysis due to very tight thresholds.
(PDF)

S12 Fig Vertical GRF results for optimization of Subject 22 at 70% PWS. B-SLIP and VPP model variations in left and right figures, respectively. Shaded region denotes standard deviation of subject’s average vertical GRF profile.
(PDF)

S13 Fig Vertical CoM and leg stiffness results for Subject 22 at 100% PWS. B-SLIP and VPP model variations in left and right figures, respectively. Markers and vertical lines denote gait events. Shaded region denotes standard deviation of average gait cycle. All template models achieved optimal solutions, but only B-SLIP (C) passed the outlier screening. Note that B-SLIP (V) and VPP (V) tracked vertical CoM within a standard deviation the entire gait cycle. This is an example of removal from analysis due to very tight thresholds.
(PDF)

S14 Fig Vertical GRF results for optimization of Subject 22 at 100% PWS. B-SLIP and VPP model variations in left and right figures, respectively. Shaded region denotes standard deviation of subject’s average vertical GRF profile.
(PDF)

S15 Fig Vertical CoM and leg stiffness results for Subject 22 at 130% PWS. B-SLIP and VPP model variations in left and right figures, respectively. Markers and vertical lines denote gait events. Shaded region denotes standard deviation of average gait cycle. All template models achieved optimal solutions, but only B-SLIP (C) passed the outlier screening. Note that B-SLIP (V) and VPP (V) tracked vertical CoM within a standard deviation the entire gait cycle. This is an example of removal from analysis due to very tight thresholds.
(PDF)

S16 Fig Vertical GRF results for optimization of Subject 22 at 130% PWS. B-SLIP and VPP model variations in left and right figures, respectively. Shaded region denotes standard deviation of subject’s average vertical GRF profile.
(PDF)

S17 Fig Linear regression analysis of GRF vs. CoM tracking error for all subject trials at 55% PWS. (PDF)

S18 Fig Linear regression analysis of GRF vs. CoM tracking error for all subject trials at PWS. (PDF)

S19 Fig Linear regression analysis of GRF vs. CoM tracking error for all subject trials at 130% PWS. (PDF)

S20 Fig Comparison of stiffness profiles generated from the optimization framework for two different values of *r*_h_. Constant stiffness and varying stiffness in left and right figures, respectively. Graphic corresponds to Subject 04 at 100% PWS. Note that for both the constant and varying stiffness models, choice of *r*_h_ does not significantly impact resulting stiffness profile from the optimization framework.
(PDF)

S21 Fig Comparison of stiffness profiles generated from the optimization framework for two different values of *r*_VP_. Constant stiffness and varying stiffness in left and right figures, respectively. Graphic corresponds to Subject 04 at 100% PWS. Note that for both the constant and varying stiffness models, choice of *r*_VP_ does not significantly impact resulting stiffness profile from the optimization framework.
(PDF)

S22 Fig Comparison of CoM trajectories based on having different temporal alignment constraints active. B-SLIP with constant stiffness and varying stiffness in left and right figures, respectively. Table corresponds to several subjects, figure corresponds to Subject 04 at 100% PWS.
(PDF)
