# Supplementary material for: Center of mass kinematic reconstruction during steady-state walking using optimized template models
Source: PLoS One. 2024 Nov 5;19(11):e0313156. doi: 10.1371/journal.pone.0313156 (PMC11537374; doi:10.1371/journal.pone.0313156)
Supplement: S1 Appendix — (PDF) [file pone.0313156.s001.pdf]

Supplementary Appendix 1

The work presented here corresponds to the geometric derivation for Eq. 5 of the manuscript for the Virtual Pivot Point (VPP) model, reintroduced in the equation below

$$\begin{aligned} \tau &= F_{\mathrm{s}} \ell \tan(\beta) \\ \tan(\beta) &= \frac{(r_{\mathrm{h}} + r_{\mathrm{VP}}) \sin(\psi)}{\ell + (r_{\mathrm{h}} + r_{\mathrm{VP}}) \cos(\psi)} \end{aligned} \tag{1}$$

where τ is the hip torque required to redirect the spring force F_{s} to the virtual point VP, ℓ is the leg length, r_{h} denotes the distance between the hip and center of mass (CoM) position, and r_{VP} denotes the distance between the CoM and VP. The angle between the pitch orientation of the trunk and the leg is denoted by ψ, and the angle through which the spring force is redirected is denoted by β.

Redirecting F_{s} towards the VP can be realized geometrically by representing τ as a force F_{N} acting orthogonal to F_{s} at the point of foot contact

$$\tau = F_{\mathrm{N}} \ell. \tag{2}$$

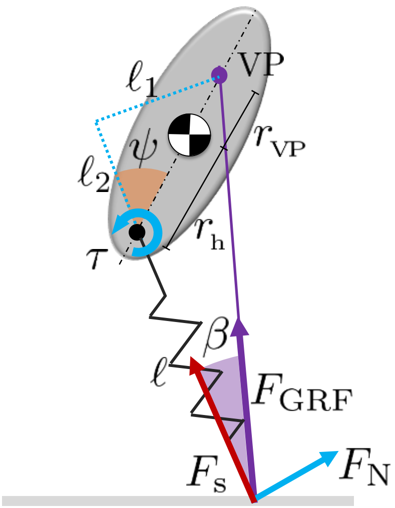

Fig 1. Labeled depiction of the VPP model.

We recognize that geometrically, Eq. (2) and the first line in Eq. (1) are equivalent, such that we don't have an unknown force to determine.

We can then use geometric relationships to further solve for the $\tan(\beta)$ term based on already known/readily calculable parameters. From Fig. 1, using the triangle formed between the line along r_{h} and r_{VP}, and the angle ψ, the values for ℓ_1 and ℓ_2 are

$$\begin{aligned} \ell_1 &= (r_{\mathrm{h}} + r_{\mathrm{VP}}) \sin(\psi) \\ \ell_2 &= (r_{\mathrm{h}} + r_{\mathrm{VP}}) \cos(\psi). \end{aligned} \tag{3}$$

With both ℓ_1 and ℓ_2 defined, we can represent $\tan(\beta)$ with known parameters as

$$\tan(\beta) = \frac{\ell_1}{\ell + \ell_2}. \tag{4}$$

Substituting Eq. (3) into Eq. (4) yields the same formulation provided in the second line of Eq. (1). The geometric relationships presented here lead to the formulation of Eq. 5 that is used when defining the dynamics of the VPP model.
