# Supplementary material for: Center of mass kinematic reconstruction during steady-state walking using optimized template models
Source: PLoS One. 2024 Nov 5;19(11):e0313156. doi: 10.1371/journal.pone.0313156 (PMC11537374; doi:10.1371/journal.pone.0313156)
Supplement: S1 Table — (PDF) [file pone.0313156.s002.pdf]

	CoM Tracking Error ϵ_C Significance (p-value)							
**Trial Speed:**	40%	55%	70%	85%	100%	115%	130%	145%
B-SLIP (C)<br>B-SLIP (V)	1.318e-04<br>***	1.964e-04<br>***	1.709e-02<br>*	4.378e-04<br>***	1.318e-04<br>***	1.318e-04<br>***	1.318e-04<br>***	9.766e-04<br>**
VPP (C)<br>VPP (V)	4.378e-04<br>***	2.931e-04<br>***	4.010e-05<br>***	1.318e-04<br>***	1.318e-04<br>***	1.318e-04<br>***	1.318e-04<br>***	6.104e-05<br>***
B-SLIP (C)<br>VPP (C)	9.588e-01	3.467e-02<br>*	3.259e-01	9.100e-02	4.848e-03<br>**	8.918e-04<br>**	9.157e-04<br>**	5.859e-03<br>*
B-SLIP (V)<br>VPP (V)	2.902e-03<br>**	1.397e-03<br>**	3.981e-04<br>***	7.662e-02	5.430e-01	3.760e-01	2.688e-02<br>*	7.240e-04<br>**

*$p<0.05$ **$p<0.005$ ***$p<0.0005$
