# Supplementary material for: Center of mass kinematic reconstruction during steady-state walking using optimized template models
Source: PLoS One. 2024 Nov 5;19(11):e0313156. doi: 10.1371/journal.pone.0313156 (PMC11537374; doi:10.1371/journal.pone.0313156)
Supplement: S2 Table — (PDF) [file pone.0313156.s003.pdf]

	GRF Matching Error ϵ_G Significance (p-value)							
**Trial Speed:**	40%	55%	70%	85%	100%	115%	130%	145%
B-SLIP (C) / B-SLIP (V)	2.502e-04 ***	3.286e-03 **	1.677e-01	4.691e-01	9.673e-04 **	1.551e-04 ***	1.318e-04 ***	9.766e-04 **
VPP (C) / VPP (V)	1.609e-03 **	1.359e-01	8.240e-02	1.474e-01	1.944e-03 **	1.551e-04 ***	1.318e-04 ***	6.105e-05 ***
B-SLIP (C) / VPP (C)	1.306e-02 *	1.762e-02 *	8.794e-02	3.639e-02 *	3.294e-02 *	3.507e-01	3.860e-03 **	3.711e-02 *
B-SLIP (V) / VPP (V)	1.410e-02 *	5.991e-04 **	5.385e-04 **	7.016e-02	5.202e-01	3.981e-01	7.240e-04 **	1.116e-03 **

*$p<0.05$ **$p<0.005$ ***$p<0.0005$
