# Supplementary material for: Center of mass kinematic reconstruction during steady-state walking using optimized template models
Source: PLoS One. 2024 Nov 5;19(11):e0313156. doi: 10.1371/journal.pone.0313156 (PMC11537374; doi:10.1371/journal.pone.0313156)
Supplement: S3 Table — (PDF) [file pone.0313156.s004.pdf]

	**Gait Event Matching Error ϵ_{t_f} Significance (p-value)**							
**Trial Speed:**	40%	55%	70%	85%	100%	115%	130%	145%
B-SLIP (C)<br>B-SLIP (V)	1.590e-01	2.491e-02<br>*	6.355e-01	7.960e-01	5.732e-01	2.688e-02<br>*	8.903e-03<br>*	2.441e-02<br>*
VPP (C)<br>VPP (V)	1.961e-01	6.838e-02	1.677e-01	2.432e-01	9.679e-01	2.422e-02<br>*	1.758e-02<br>*	6.714e-03<br>*
B-SLIP (C)<br>VPP (C)	5.695e-01	7.112e-01	2.618e-02<br>*	6.874e-01	8.405e-01	9.296e-02	2.080e-02<br>*	1.953e-03<br>**
B-SLIP (V)<br>VPP (V)	5.197e-01	1.024e-01	1.000e-02<br>*	6.874e-01	7.151e-01	5.732e-01	3.639e-02<br>*	1.147e-03<br>**

*p<0.05 **p<0.005 ***p<0.0005
