# Supplementary material for: Center of mass kinematic reconstruction during steady-state walking using optimized template models
Source: PLoS One. 2024 Nov 5;19(11):e0313156. doi: 10.1371/journal.pone.0313156 (PMC11537374; doi:10.1371/journal.pone.0313156)
Supplement: S4 Table — (PDF) [file pone.0313156.s005.pdf]

CoM Tracking Error ϵ_C Standard Deviation Significance (p-value)								
**Trial Speed:**	40%	55%	70%	85%	100%	115%	130%	145%
B-SLIP (C)<br>B-SLIP (V)	1.976e-21<br>***	1.913e-30<br>***	3.141e-11<br>***	7.834e-09<br>***	1.609e-18<br>***	5.401e-29<br>***	2.898e-26<br>***	3.224e-18<br>***
VPP (C)<br>VPP (V)	6.687e-18<br>***	2.007e-31<br>***	3.164e-05<br>***	1.328e-04<br>***	8.751e-14<br>***	1.316e-26<br>***	8.900e-27<br>***	4.170e-20<br>***
B-SLIP (C)<br>VPP (C)	2.142e-01	6.289e-03<br>*	2.914e-23<br>***	1.094e-02<br>*	8.277e-02	4.110e-01	6.848e-01	3.947e-01
B-SLIP (V)<br>VPP (V)	3.641e-01	7.617e-02	8.283e-15<br>***	6.869e-01	7.946e-01	2.720e-01	9.268e-01	7.168e-01

*$p<0.05$ **$p<0.005$ ***$p<0.0005$
