# Supplementary material for: Center of mass kinematic reconstruction during steady-state walking using optimized template models
Source: PLoS One. 2024 Nov 5;19(11):e0313156. doi: 10.1371/journal.pone.0313156 (PMC11537374; doi:10.1371/journal.pone.0313156)
Supplement: S5 Table — (PDF) [file pone.0313156.s006.pdf]

	**GRF Matching Error ϵ_G Standard Deviation Significance (p-value)**							
**Trial Speed:**	40%	55%	70%	85%	100%	115%	130%	145%
B-SLIP (C)<br>B-SLIP (V)	4.746e-06<br>***	8.038e-06<br>***	8.207e-02	6.057e-01	4.589e-06<br>***	3.152e-08<br>***	1.776e-04<br>***	1.462e-04<br>***
VPP (C)<br>VPP (V)	1.348e-02<br>*	1.046e-04<br>***	2.504e-01	5.936e-01	3.382e-05<br>***	2.023e-04<br>***	6.583e-04<br>**	9.630e-02
B-SLIP (C)<br>VPP (C)	1.580e-02<br>*	8.644e-02	4.008e-05<br>***	1.452e-01	8.014e-01	4.018e-01	8.098e-01	2.239e-01
B-SLIP (V)<br>VPP (V)	6.562e-01	3.846e-01	1.342e-06<br>***	6.674e-01	8.591e-01	2.170e-03<br>**	5.371e-01	2.453e-01

*$p<0.05$ **$p<0.005$ ***$p<0.0005$
