# Supplementary material for: Center of mass kinematic reconstruction during steady-state walking using optimized template models
Source: PLoS One. 2024 Nov 5;19(11):e0313156. doi: 10.1371/journal.pone.0313156 (PMC11537374; doi:10.1371/journal.pone.0313156)
Supplement: S6 Table — (PDF) [file pone.0313156.s007.pdf]

**Gait Event Matching Error ϵ_{t_f} Standard Deviation Significance (p-value)**								
**Trial Speed:**	40%	55%	70%	85%	100%	115%	130%	145%
B-SLIP (C)<br>B-SLIP (V)	9.638e-01	6.368e-01	4.768e-02<br>*	2.344e-01	7.380e-01	1.180e-01	9.106e-01	2.047e-01
VPP (C)<br>VPP (V)	1.296-03<br>**	9.831e-01	5.463e-01	2.504e-01	5.845e-01	8.808e-01	4.729e-02<br>*	6.226e-01
B-SLIP (C)<br>VPP (C)	9.017e-01	4.440e-01	7.506e-01	6.508e-02	9.423e-01	4.839e-01	8.764e-01	7.495e-01
B-SLIP (V)<br>VPP (V)	7.316e-04<br>**	7.547e-01	5.429e-02	6.318e-01	8.830e-01	4.877e-01	5.933e-02	1.511e-01

*$p<0.05$ **$p<0.005$ ***$p<0.0005$
