# Supplementary material for: Center of mass kinematic reconstruction during steady-state walking using optimized template models
Source: PLoS One. 2024 Nov 5;19(11):e0313156. doi: 10.1371/journal.pone.0313156 (PMC11537374; doi:10.1371/journal.pone.0313156)
Supplement: S7 Table — (PDF) [file pone.0313156.s008.pdf]

Gait Event Duration Comparison					
**Subject**	**Speed (m/s)**	**DS1 (s)**	**SS1 (s)**	**DS2 (s)**	**SS2 (s)**
Subj. 1	1.21	0.1354	0.4061	0.1472	0.3780
Subj. 19		0.1456	0.3541	0.1600	0.3525
Subj. 13	1.02	0.1462	0.4179	0.1493	0.4209
Subj. 24		0.1641	0.3608	0.1620	0.3600
Subj. 6	1.28	0.1429	0.3705	0.1454	0.3794
Subj. 20		0.1430	0.3494	0.1516	0.3365
Subj. 4	0.52	0.3323	0.5252	0.3386	0.5144
Subj. 5		0.2864	0.5008	0.3285	0.4764
