# Supplementary material for: Center of mass kinematic reconstruction during steady-state walking using optimized template models
Source: PLoS One. 2024 Nov 5;19(11):e0313156. doi: 10.1371/journal.pone.0313156 (PMC11537374; doi:10.1371/journal.pone.0313156)
Supplement: S8 Table — (PDF) [file pone.0313156.s009.pdf]

Effect of Mass Inertia Parameter on Optimization Results (At PWS)

Subject	Weight (kg)	Inertia J (kg m^2)	ϵ_{C}	ϵ_{G}	$\epsilon_{\mathrm{t_f}}$
Subj. 2	52.9	4.58	0.0065	0.1454	0.0130
		6.41	0.0065	0.1458	0.0131
Subj. 4	61.05	4.58	0.0083	0.1060	0.0130
		6.41	0.0083	0.1076	0.0124
Subj. 7	71.75	4.58	0.0083	0.1155	0.0198
		6.41	0.0083	0.1184	0.0198
Subj. 13	95.4	4.58	0.0082	0.0745	0.0195
		6.41	0.0082	0.0768	0.0190
