# Supplementary material for: Center of mass kinematic reconstruction during steady-state walking using optimized template models
Source: PLoS One. 2024 Nov 5;19(11):e0313156. doi: 10.1371/journal.pone.0313156 (PMC11537374; doi:10.1371/journal.pone.0313156)
Supplement: S4 Fig — B-SLIP and VPP model variations in left and right figures, respectively. Shaded region denotes standard deviation of subject’s average vertical GRF profile. (PDF) [file pone.0313156.s013.pdf]

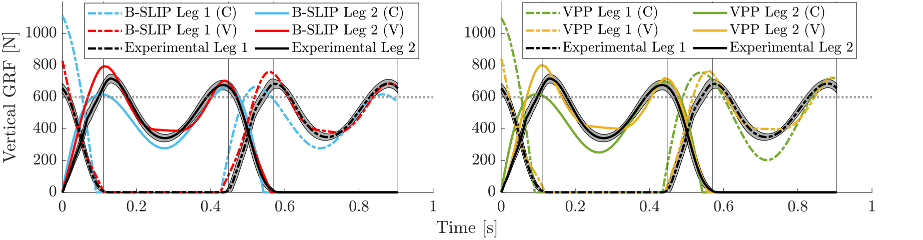

Fig S4. Vertical GRF results for optimization of Subject 4 at 130% PWS. B-SLIP and VPP model variations in left and right figures, respectively. Shaded region denotes standard deviation of subject's average vertical GRF profile.
