# Supplementary material for: Center of mass kinematic reconstruction during steady-state walking using optimized template models
Source: PLoS One. 2024 Nov 5;19(11):e0313156. doi: 10.1371/journal.pone.0313156 (PMC11537374; doi:10.1371/journal.pone.0313156)
Supplement: S9 Fig — B-SLIP and VPP model variations in left and right figures, respectively. Markers and vertical lines denote gait events. Shaded region denotes standard deviation of average gait cycle. All template models achieved optimal solutions and passed the outlier screening for this subject-trial pair. (PDF) [file pone.0313156.s018.pdf]

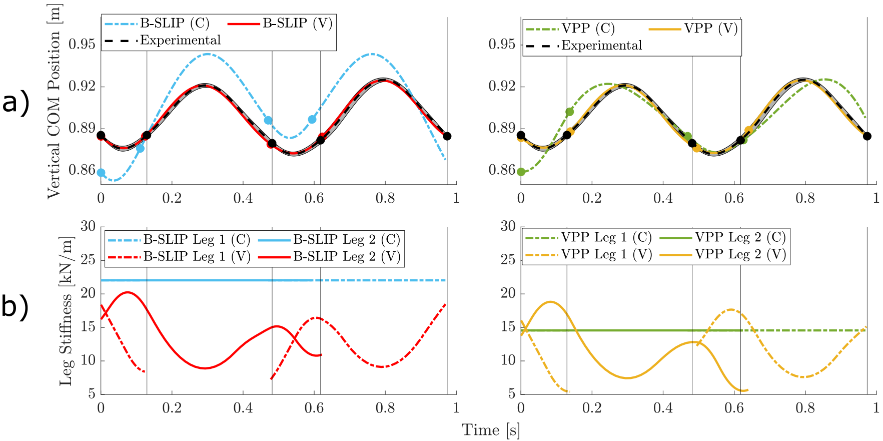

Fig S9. Vertical CoM and leg stiffness results for Subject 13 at 130% PWS. B-SLIP and VPP model variations in left and right figures, respectively. Markers and vertical lines denote gait events. Shaded region denotes standard deviation of average gait cycle. All template models achieved optimal solutions and passed the outlier screening for this subject-trial pair.
