# Supplementary material for: Center of mass kinematic reconstruction during steady-state walking using optimized template models
Source: PLoS One. 2024 Nov 5;19(11):e0313156. doi: 10.1371/journal.pone.0313156 (PMC11537374; doi:10.1371/journal.pone.0313156)
Supplement: S15 Fig — B-SLIP and VPP model variations in left and right figures, respectively. Markers and vertical lines denote gait events. Shaded region denotes standard deviation of average gait cycle. All template models achieved optimal solutions, but only B-SLIP (C) passed the outlier screening. Note that B-SLIP (V) and VPP (V) tracked vertical CoM within a standard deviation the entire gait cycle. This is an example of removal from analysis due to very tight thresholds. (PDF) [file pone.0313156.s024.pdf]

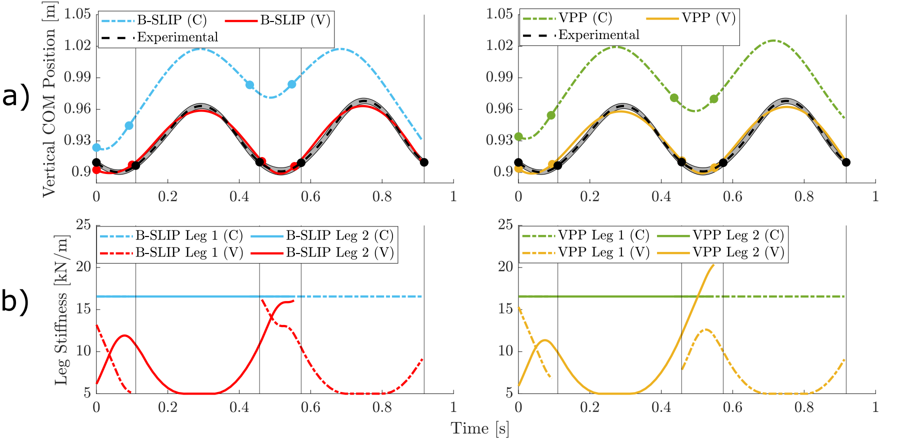

Fig S15. Vertical CoM and leg stiffness results for Subject 22 at 130% PWS. B-SLIP and VPP model variations in left and right figures, respectively. Markers and vertical lines denote gait events. Shaded region denotes standard deviation of average gait cycle. Both constant stiffness models failed to achieve an optimal solution. Note that both varying stiffness models tracked vertical CoM within a standard deviation the entire gait cycle, yet did not pass the outlier screening. This is an example of removal from analysis due to very tight thresholds.
