# Supplementary figures and images for: Center of mass kinematic reconstruction during steady-state walking using optimized template models
Source: PLoS One. 2024 Nov 5;19(11):e0313156. doi: 10.1371/journal.pone.0313156 (PMC11537374; doi:10.1371/journal.pone.0313156)

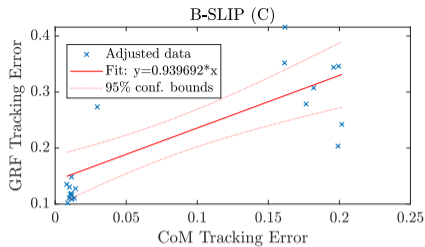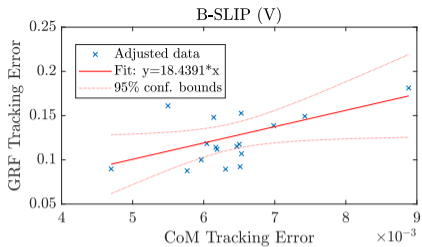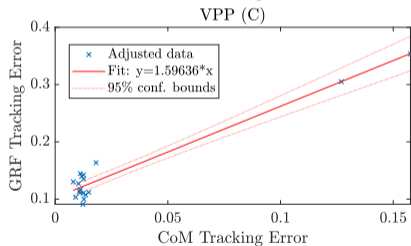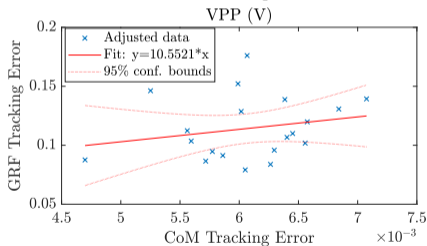

**Fig S17. Linear regression analysis of GRF vs. CoM tracking error for all subject trials at 55% PWS.**

Supplement: S17 Fig — (PDF) [file pone.0313156.s026.pdf]

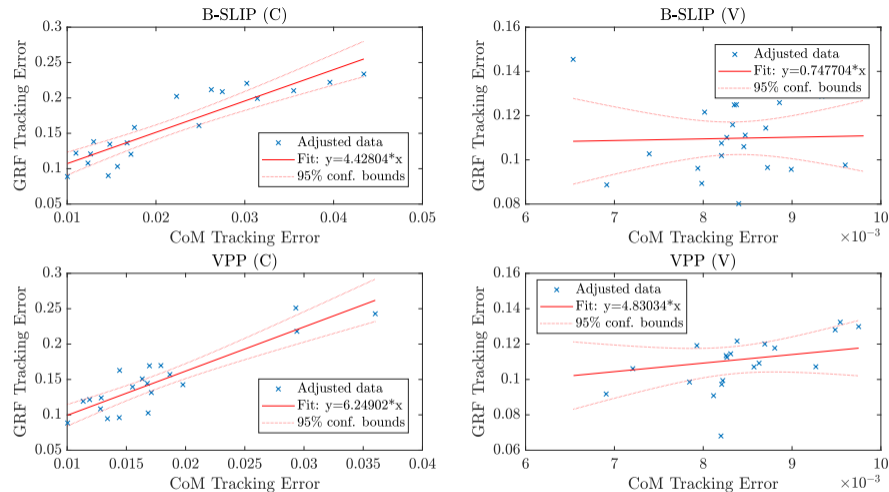

**Fig S18. Linear regression analysis of GRF vs. CoM tracking error for all subject trials at PWS.**

Supplement: S18 Fig — (PDF) [file pone.0313156.s027.pdf]

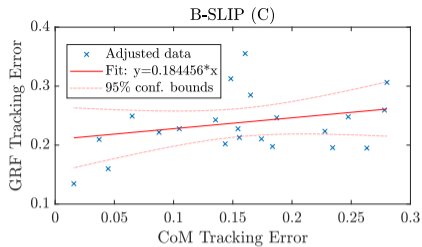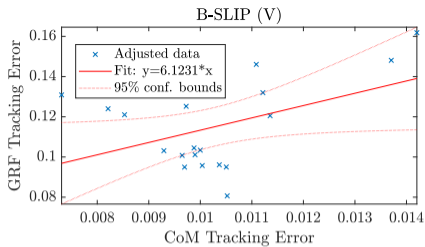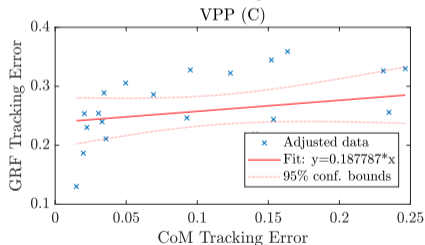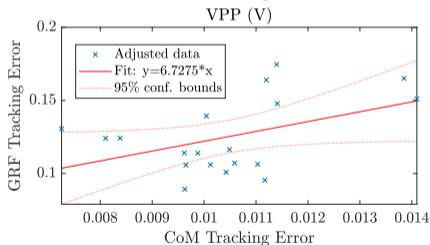

**Fig S19. Linear regression analysis of GRF vs. CoM tracking error for all subject trials at 130% PWS.**

Supplement: S19 Fig — (PDF) [file pone.0313156.s028.pdf]
