# Supplementary material for: Center of mass kinematic reconstruction during steady-state walking using optimized template models
Source: PLoS One. 2024 Nov 5;19(11):e0313156. doi: 10.1371/journal.pone.0313156 (PMC11537374; doi:10.1371/journal.pone.0313156)
Supplement: S20 Fig — Constant stiffness and varying stiffness in left and right figures, respectively. Graphic corresponds to Subject 04 at 100% PWS. Note that for both the constant and varying stiffness models, choice of rh does not significantly impact resulting stiffness profile from the optimization framework. (PDF) [file pone.0313156.s029.pdf]

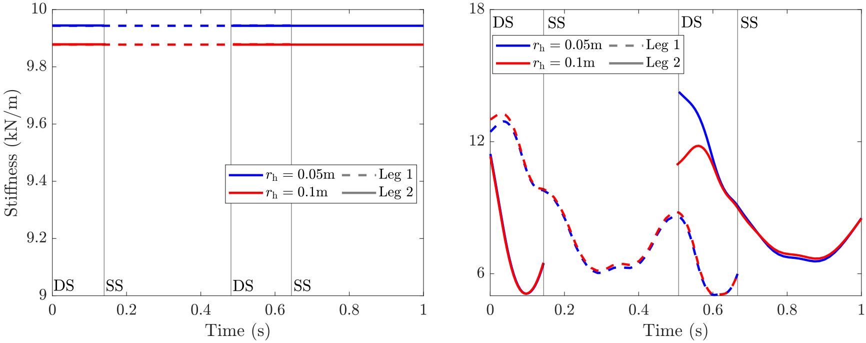

Fig S20. Comparison of stiffness profiles generated from the optimization framework for two different values of r_h. Constant stiffness and varying stiffness in left and right figures, respectively. Graphic corresponds to Subject 04 at 100% PWS. Note that for both the constant and varying stiffness models, choice of r_h does not significantly impact resulting stiffness profile from the optimization framework.
