# Supplementary material for: Center of mass kinematic reconstruction during steady-state walking using optimized template models
Source: PLoS One. 2024 Nov 5;19(11):e0313156. doi: 10.1371/journal.pone.0313156 (PMC11537374; doi:10.1371/journal.pone.0313156)
Supplement: S22 Fig — B-SLIP with constant stiffness and varying stiffness in left and right figures, respectively. Table corresponds to several subjects, figure corresponds to Subject 04 at 100% PWS. (PDF) [file pone.0313156.s031.pdf]

	**Subj. 2**	**Subj. 4**	**Subj. 7**	**Subj. 13**
**Constant**	Gait Event Matching Error ϵ_{t_f} (s)			
All Constraints	0.0206	0.0212	0.0124	0.0110
Gait Duration^1	0.0763	0.1379	0.0988	0.0848
Gait Duration^2	0.0715	0.0696	0.0733	0.1964
No Constraints	0.1857	0.1958	0.2445	0.2748
**Varying**	Gait Event Matching Error ϵ_{t_f} (s)			
All Constraints	0.0028	0.0184	0.0148	0.0128
Gait Duration	0.0037	0.0106	0.0152	0.3049
No Constraints	0.1830	0.1903	0.2051	0.1513

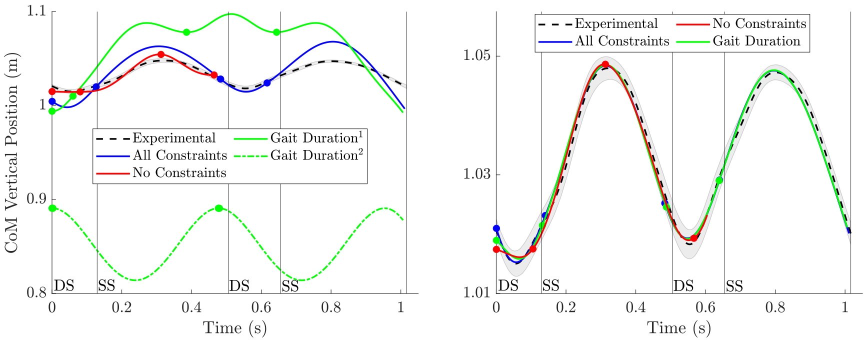

Fig S22. Comparison of CoM trajectories based on having different temporal alignment constraints active. B-SLIP with constant stiffness and varying stiffness in left and right figures, respectively. Table corresponds to several subjects, figure corresponds to Subject 04 at 100% PWS. “All Constraints” means that constraints for phase duration (i.e., SS and DS) and total gait duration were active. “Gait Duration” means that only total gait duration was active. “No Constraints” means no temporal alignment constraints were active. Note that the constant stiffness model was sensitive to initial guess on leg stiffness, so Gait Duration^1 was initialized at $k_{\text{leg}} = 20$kN/m, while Gait Duration^2 was initialized at $k_{\text{leg}} = 6$ kN/m. The constant stiffness failed to appropriately track the experimental CoM data if any temporal alignment constraints were removed. The varying stiffness model was able to still accurately track experimental CoM data even with phase duration constraints removed. Neither model achieved appropriate tracking when all temporal alignment constraints were removed. The table above highlights the impact of temporal alignment constraints on the performance for both constant and varying stiffness models across several subjects. Except for Subject 13, all subjects saw consistently better gait event duration matching from the varying stiffness model compared to the constant stiffness model when removing one or more temporal alignment constraints.
